# Reduced linguistic coherence in psychosis defies semantic similarity accounts and relates to altered large-scale cortical hierarchy

**DOI:** 10.1038/s41598-026-39025-1

**Published:** 2026-02-08

**Authors:** Rui He, Radosław Grodzki, Nihal Altay, Benjamin Aston, Maria Francisca Alonso-Sánchez, Philipp Homan, Iris Sommer, Lena Palaniyappan, Wolfram Hinzen

**Affiliations:** 1 Grammar and Cognition Lab, Department of Translation & Language Sciences, Universitat Pompeu Fabra, Carrer Roc Boronat, 138, Barcelona, 08018 Spain; 2 Department of Adult Psychiatry and Psychotherapy, University of Zurich, Zurich, Switzerland; 3 Escuela de Fonoaudiología, CIDCL, Universidad de Valparaíso, Valparaíso, Chile; 4 Neuroscience Center Zurich, University of Zurich and ETH Zurich, Zurich, Switzerland; 5 Department of Neuroscience, University Medical Center Groningen, Antoni Deusinglaan 2, room 117, Groningen, The Netherlands; 6 Department of Psychiatry, Douglas Mental Health University Institute, McGill University, Montreal, QC Canada; 7 Department of Medical Biophysics, Schulich School of Medicine and Dentistry, Western University, London, ON Canada; 8 Robarts Research Institute, Schulich School of Medicine and Dentistry, Western University, London, ON Canada; 9 Institut Català de Recerca i Estudis Avançats (ICREA), Barcelona, Spain

**Keywords:** Schizophrenia, Coherence, Language models, Natural language processing, Functional gradient, Computational biology and bioinformatics, Neuroscience, Psychology, Psychology

## Abstract

Coherence in speech is clinically significant in mental disorders but remains difficult to quantify. We tested the widely-held assumption that semantic similarity metrics derived from large language models capture human-rated coherence. Across three large neurotypical datasets in different languages, semantic similarity failed to correlate with human ratings, while six other metrics, especially the probability-based metrics, showed significant but weak correlations. In an additional English dataset of 94 individuals, including healthy controls and patients with schizophrenia spectrum disorders (SSD), speech coherence was reduced in SSD. Incoherence in the drug-naïve first-episode samples related to altered whole-brain intrinsic functional gradients, and to the probabilistic metric of perplexity in speech. Together, these findings call into question semantic similarity as a proxy for coherence, motivate greater emphasis on probabilistic predictability measures for evaluating coherence, and substantiate the perspective of spontaneous speech as an overt readout of an alteration of hierarchical cortical organization in schizophrenia.

**Supplementary Information:**

The online version contains supplementary material available at 10.1038/s41598-026-39025-1.

## Introduction

Language as freely produced in discourse or conversation brings together a number of cognitive functions in an integrated fashion: word forms and meanings as stored in declarative memory have to be retrieved, grammatical structure has to be built using procedural memory, speech needs to match affect and perceptions, a discourse plan has to be pursued and monitored, and the mental state of the interlocutor has to be represented. This complex interplay of cognitive functions across the whole brain makes language a likely sensitive marker of cognitive dysfunction. Yet quantifying language function poses challenges. This, in particular, applies to quantifying coherence at the discourse level, including the loss of such coherence in schizophrenia spectrum disorders (SSD), characterized by Eugen Bleuler as ‘loosening of associations’^1^. A decrease in averaged semantic similarity between consecutive words using latent semantic analysis has been used to quantify this phenomenon since the pioneering work of Elvevåg et al. in 2007^2^. Since then, measuring semantic similarity with language models (LMs) to detect incoherence in speech in psychosis has thrived for two decades, the leading intuition being that loosening corresponds to a ‘widening’ of the semantic space (larger semantic distances crossed)^3–7^.

However, there is, in fact, little a priori or empirical reason to believe that semantic similarity between adjacent words or sentences reliably indicates the coherence of a text. To give a striking example, *A fox jumped over the tree* is more coherent than its scrambled version *fox tree jumped over the a*, yet the mean semantic similarity between the adjacent words of the former sentence is 0.160, using the English fastText model, while that of the latter is higher (0.194). Even when using semantic similarity scores as derived from contextual models, which are sensitive to grammar^8^, we fail to capture the loss of coherence in the scrambled sentence: the scores for BERT are 0.465 vs. 0.512, and 0.981 vs. 0.982 for GPT-2. Moreover, to our best knowledge, the assumption of a relation between semantic similarity and coherence has not been empirically tested thoroughly, and it has recently been empirically challenged. Thus, several studies have found *in*creased semantic similarity between consecutive words or sentences in patients with SSD as compared to healthy individuals, conceptualized as indicating a ‘shrunk’ semantic space^9–12^, contradicting the traditional expectation of a ‘wider’ semantic space. Heterogeneity in semantic similarity metrics has also been observed across datasets in different languages^13^. In addition, changes in semantic similarity are not specific to SSD, but are also reported in other brain disorders such as bipolar disorder^14^ and Alzheimer’s disease^15^. A conceptual shift has been suggested from using semantic similarity as a marker of coherence to viewing it as a quantitative metric of how speakers traverse conceptual semantic space during speech^10,16^, leaving open how alterations in such patterns might connect to human ratings of coherence.

This study aimed to directly address this connection. Coherence is a crucial linguistic property that pertains to the relationships among discourse units (e.g., words and utterances), ensuring that the discourse is logically consistent, bound to a central topic, and easily understandable^17^. Previous studies have modeled coherence with entity relations^18^, lexical semantic similarity graphs^19^, sentence semantic similarity graphs^20^, semantic focus^20^, and semantic surprisal^21^. These studies suggest that features of the semantic space can serve as markers for discourse coherence, but it has remained unclear which of them should be specifically targeted. Going beyond simply averaging semantic similarity values, previous work has expanded the feature set by introducing comprehensive descriptors of distributions beyond averaged scores, including extrema and skewness; graph-theoretical features to approximate the topology of the high-dimensional space; and wave-based dynamic features that capture how semantic similarity scores evolve over narrative time^22–24^.

Additionally, potential computational measures of coherence should not be confined solely to semantic similarity metrics. Recent advances in language modeling have approached language as a prediction task: each token from the vocabulary has a specific probability of occurring in each position in a sentence, which can be quantified based on the preceding contexts by generative language models. Using probability-based metrics in addition to semantic similarity ones, such as semantic surprisal^21^ and perplexity^25^, could yield additional insights into the linguistic properties of language and coherence. Recently, Sharpe et al. ^26^ found that the predictability of a word given its preceding context during speech production distinguished patients with positive formal thought disorder, who were specifically insensitive to global (long-distance) versus local linguistic context. Semantic structure in spontaneous speech may not only offer a window into formal aspects of the disorganization of language in formal thought disorder, but reveal aspects of the content of thought as conveyed in language as well. Thus, Momeni and Raghibdoust found strong correlations between human-rated speech coherence and delusions^27^.

The clinical significance of speech coherence suggests that it could be an important readout of neurofunctional changes in brain diseases. As coherence reflects an integration across a large range of brain functions and associated networks – from semantic and working memory to attention, executive control, and theory of mind – it makes sense to approach coherence as a whole-brain effect requiring integration of information across different networks. Functional organization and integration across the cortex can be summarized in a lower-dimensional fashion through gradients of functional connectivity across the cortical surface. The principal cortical gradient is interpretable as a continuous transition along a hierarchy from low-level and externally-oriented visual and somatomotor networks (VN and SMN), to the high-level and internally-directed DMN. This gradient thereby approximates thought itself as a gradual abstraction from lower-level features^28^. In SSD, gradient analyses have previously revealed changes in both whole-cortical gradients and those within a more narrow semantic network comprising the left default mode and limbic regions^24^. Gradient dispersion in the principal gradient quantifies the difference in mean gradient values between any two functional networks, and was compressed between VN and DMN in FEP and within the semantic network, while the dispersion between SMN and DMN was inflated^24^.

Interestingly, discourse coherence, as measured by averaged semantic similarity between consecutive word embeddings or between each word embedding and the overall “topic” as obtained from the averaged word embeddings, has recently been found to correlate with neural activity in the DMN, prefrontal, and sensory regions^5,29^, and with large-scale cortical gradients of functional connectivity as mentioned above^30^. Other studies have shown that during comprehension, a wide range of cortical regions is less activated when processing incoherent speech stimuli, particularly in DMN regions such as temporal cortex, inferior frontal gyrus, middle temporal gyrus, dorsal medial prefrontal cortex, and fusiform gyrus^31–34^. These findings suggest that coherent speech engages a wide array of brain regions organized across a cortical hierarchy, which also aligns with current views of real-world language use as having a whole-brain distribution and requiring integration across a large range of networks^35^.

**Fig. 1:**
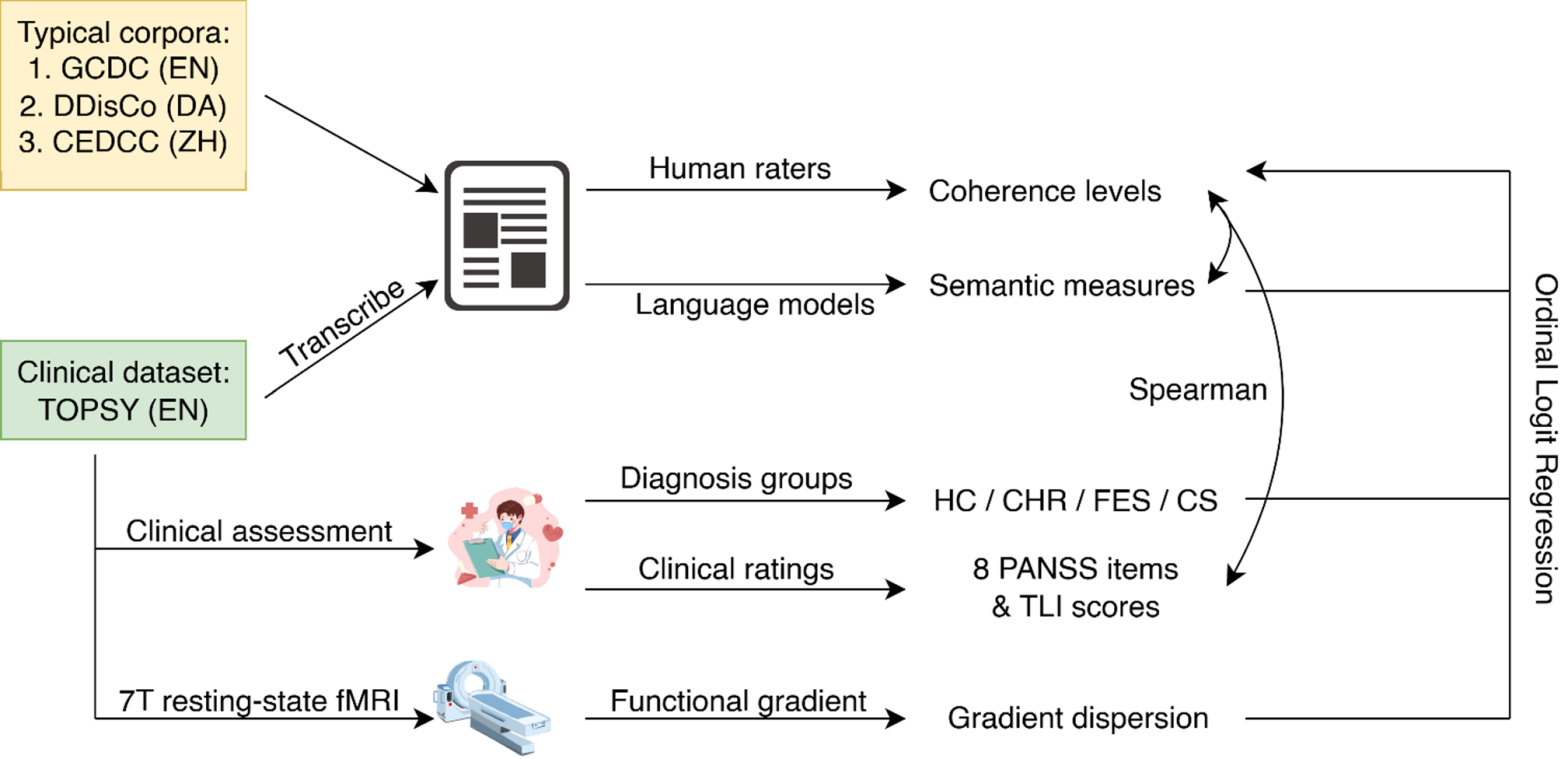
Workflow of the study.

In this study, we firstly examined whether computational semantic measures derived from LMs can reliably indicate speech coherence as assessed in large corpora from the general population. To this end, we employed three datasets across three different languages and expanded standardly used consecutive semantic similarity analysis into a broader set of measures, including probabilistic ones, for evaluating discourse coherence. In a second step, we aimed to confirm, using the same criteria, that human-rated coherence does indeed differ between groups across various sections of the SSD spectrum and controls. In this same clinical dataset, we retested the effects of the identified semantic and probabilistic measures on speech coherence, and how they interacted with the classification of groups within SSD. Finally, we investigated the relations between coherence, semantic similarity, and the cortical hierarchy of intrinsic functional connectivity, in a subset of the dataset including controls and first-episode patients, using 7T resting-state fMRI. See Fig. 1 for the workflow of this study.

We found that, out of a total of 131 semantic and probabilistic measures, only 6 yielded significant but weak correlations with human-rated coherence consistent across three large crowdsourced datasets, with the metrics using averaged semantic similarity between words failing to show such correlations. We then confirmed a gradient of decline in human-rated discourse coherence relative to controls, ranging from clinical high risk to chronic to first episode of schizophrenia, which correlated with clinical scores of delusions, unusual thought content, and disorganization of thought. Speech incoherence in FEP was moderated by word-level perplexity, while speech incoherence in chronic schizophrenia (CS) was moderated by skewness of the sentence similarity distribution. Finally, greater dispersion in the principal cortical gradient was positively related to coherence. Together these findings confirm the widely held assumption that speech in schizophrenia across different symptom profiles is less coherent, with an associated neural correlate in a whole-cortex intrinsic connectivity pattern, but they challenge the widely held assumption that altered semantic similarity between words may reflect a decline in coherence.

## Results

### Semantic and probabilistic correlates of coherence

To assess correlations between semantic and probabilistic metrics and human-rated coherence of texts, we utilized existing coherence ratings from three datasets from the general population: 4800 English texts from four different resources into the Grammarly Corpus of Discourse Coherence (GCDC)^36^, 1002 Reddit and Wiki texts in Danish as in the DDisCo dataset^37^, and 500 essays in Chinese as in the CEDCC dataset^38^. Expert ratings on coherence were made on a 3-point scale from 1 (low coherence) to 3 (high coherence) based on the principle: “a text with high coherence is easy to understand, well-organized, and contains only details that support the main point of the text. A text with low coherence is difficult to understand, not well organized, or contains unnecessary details. Try to ignore the effects of grammar or spelling errors when assigning a coherence rating”^36^. We comparatively introduced different language models to represent meaning at different levels, where context-free models such as fastText represent the de-contextualized conceptual semantic space, contextual models such as BERT represent meaning at a level that is sensitive to context and grammar, and sentence models represent meaning at sentence level. Empirical evidence has shown distinct patterns of changes with different models in SSD^10^.

Our comprehensive set of semantic measures comprised semantic similarity-related ones and their statistical distribution (e.g. averaged semantic similarity between consecutive meaningful units (first-order mean – MeanK1)). We also treated semantic similarities between consecutive units (e.g., words or sentences) as an array with temporal order, from the first binary pair to the last binary pairs of meaningful units, and selected statistical descriptors of the resulting wave function, capturing the data distribution and also dynamics of semantic similarities over time (e.g., autocorrelation). Furthermore, meaningful units were represented as graph nodes, with semantic similarity serving as edges, to capture small-world properties via the integration and segregation of nodes. We also approximated topics as the averaged embedding across meaning units (i.e. the centroids), computed the similarity of each embedding to the centroid, and extracted statistical descriptors as previously described. Finally, two probability-based metrics were added. Word-level perplexity (Word_PPL) is defined as the exponential of the average negative log-likelihood of a sequence of words, using Mistral models^39^. The sentence-level perplexity (BERT_NSP_PPL) was defined as the exponential of the average negative log-likelihood of a sequence of sentences, based on the probability scores from next sentence prediction (NSP) by BERT models. For details of the resulting 131 measures see *Methods* (*Feature extraction*).

**Fig. 2:**
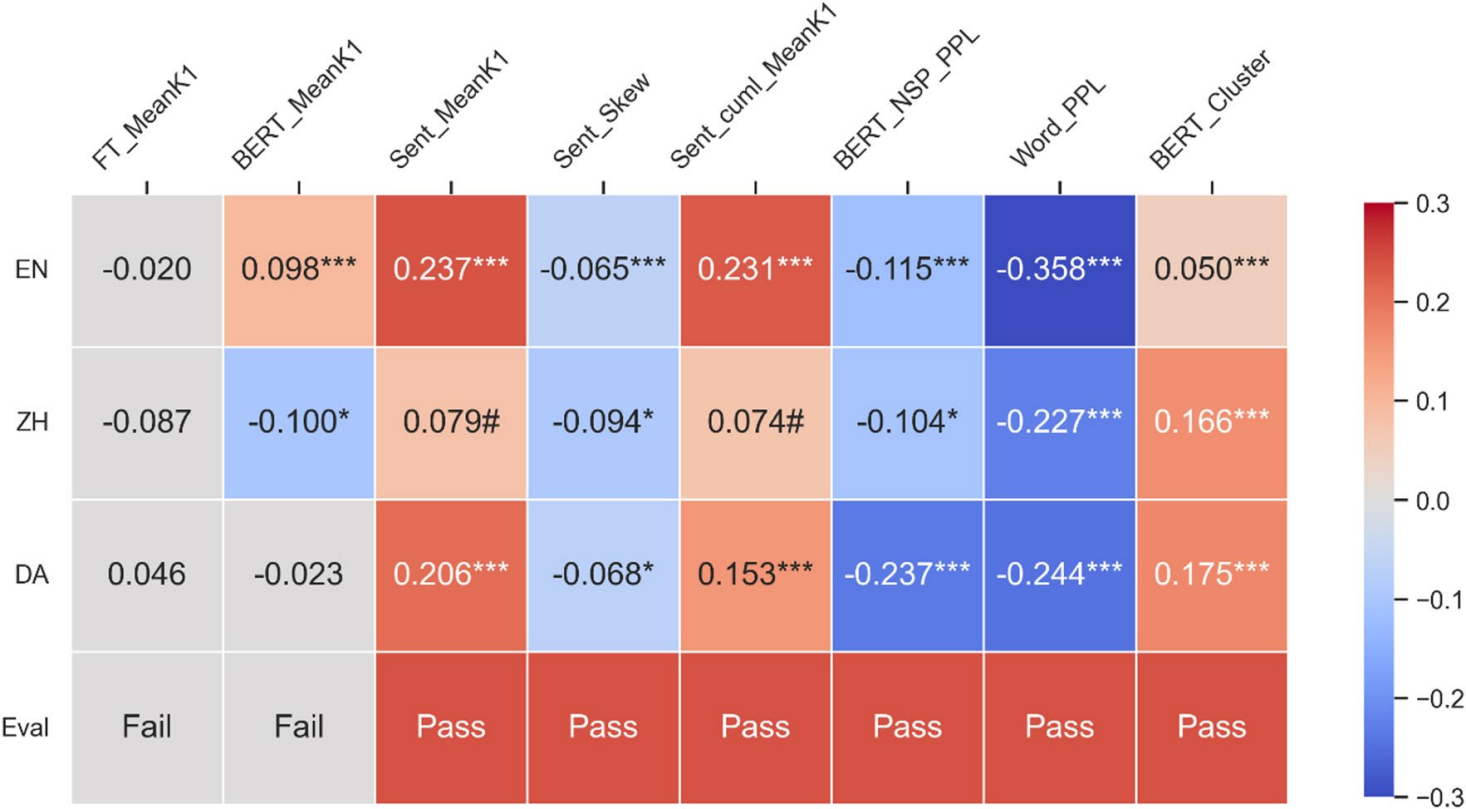
Semantic correlates of coherence in the three datasets from the general population. In the heatmap, the first row indicates the results in the English data (EN), followed by Chinese data (ZH) and Danish (DA) data. Columns refer to semantic measures that either are related to averaged semantic similarity measures (first two columns), or else the evaluation (other columns). Numbers in the cells are Spearman’s correlation coefficients between each measure and coherence in the corresponding dataset. The last row shows the evaluation results, as Pass, Uncertain (Unc), or Fail. ***q < 0.001, **q < 0.01, *q < 0.05, ^#^*q* < 0. Only correlations with significance levels over 0.1 are highlighted, with warm colors for positive ones and cold colors for negative ones. The eight variables are: averaged semantic similarity between consecutive lexical categories from fastText models (FT_MeanK1), averaged semantic similarity between consecutive tokens from BERT models (BERT_MeanK1), averaged semantic similarity between consecutive sentences (Sent_MeanK1), skewness of the distribution of sentence similarity scores (Sent_Skew), averaged semantic similarity between sentences and their cumulative centroid (Sent_cuml_MeanK1), perplexity based on next sentence prediction probability from BERT models (BERT_NSP_PPL), perplexity based on next token prediction probability from Mistral models (Word_PPL), and averaged clustering coefficients of BERT-based semantic graphs (BERT_Cluster).

Results of Spearman’s ranked correlations between every measure and human coherence ratings in each of the three crowdsourced datasets are shown in Fig. 2. The *p* values corrected per feature across three languages using false discovery rate (FDR) are reported as *q* values. Statistical significance was recognized at the level of 0.05 after correction. For every feature, we assigned the value “Pass” if the correlation coefficients in three languages shared identical signs and significant correlations were observed in three languages, or significant correlations were observed in two languages and nearly significant correlations were observed in the third language (*q* < 0.1). Six measures “passed” the evaluation: decreases in coherence correlated with less clusters in the BERT-based semantic graph, lower mean semantic similarity among sentences, more positively skewed distribution of sentence similarity scores, sentences deviating further from the cumulative centroid, and lower predictability of both next sentences and next words. These correlations, however, were all weak (*ρ* < 0.3, except for a slightly higher word-level perplexity in the English dataset (*ρ* = -0.358)). In addition, we classified those features as “Uncertain (Unc)” where significant correlations were only observed in two languages, and the coefficients for those two languages were identical. Results on the whole feature set, especially these 28 uncertain variables, are reported in Supplementary Information (SI).

Given the extensive use of the variable of averaged semantic similarity between consecutive words and a measure of coherence in previous studies, we also report results on these in Fig. 2, for completeness. Correlations between the non-contextual (purely conceptual) semantic measures as based on fastText, which were the weakest, did not obtain significance. Correlations for BERT-based averaged contextual semantic similarity were stronger but also heterogenous: significant positive correlations between BERT-based averaged semantic similarity were observed in English data, but significant negative correlations in Chinese and insignificant negative correlations in Danish. A fair conclusion would be that these results do not support the thinking that averaged semantic similarity between consecutive words indicates coherence.

### Coherence in SSD and its relation to clinical symptoms

**Fig. 3:**
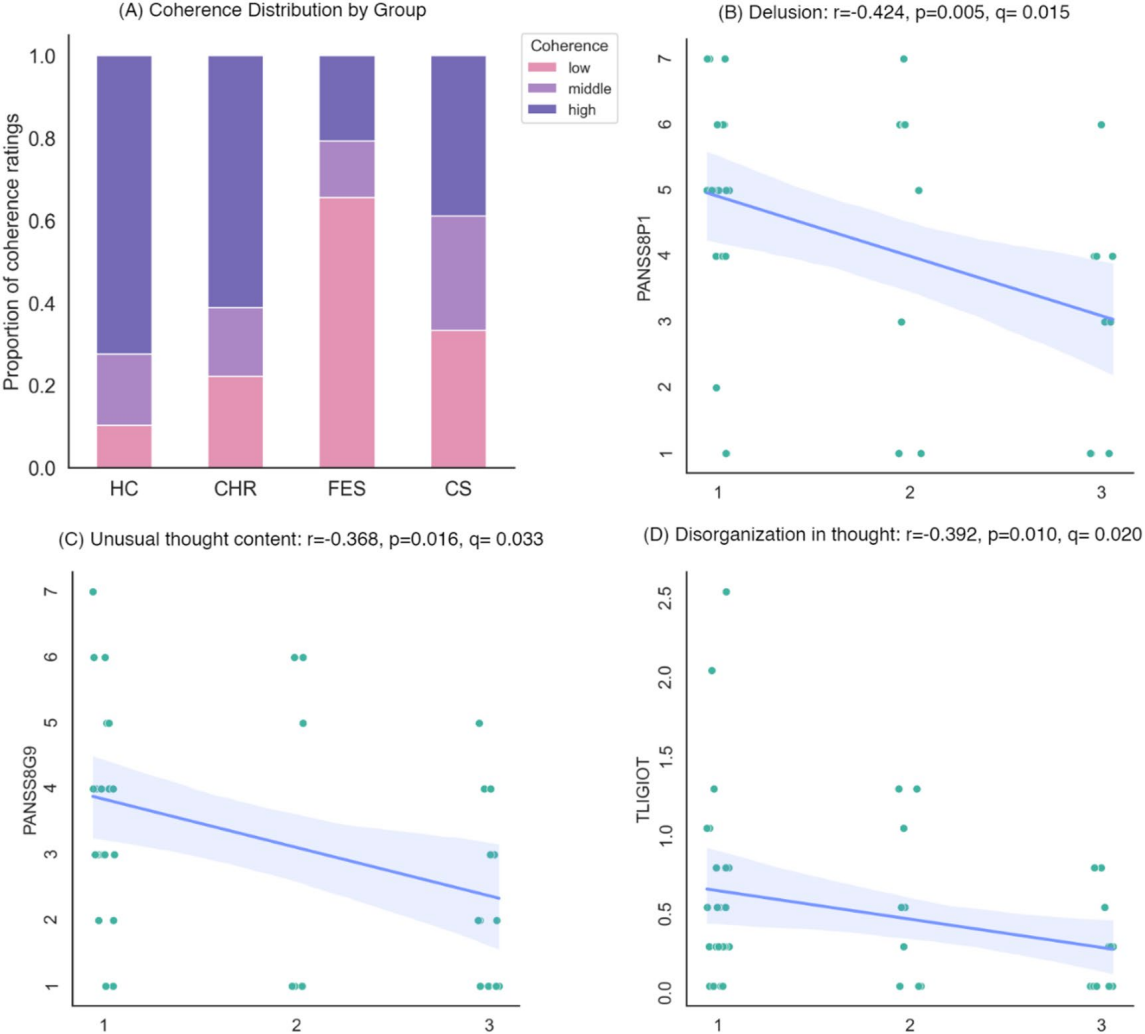
Group differences and correlation with clinical scores. (**A**) Distribution of coherence ratings across the four groups healthy control (HC), clinical high risk (CHR), first-episode psychosis (FEP), and chronic schizophrenia (CS). Coherence Rating: 1 = Low, 2 = Medium, 3 = High, same for panels (**B**–**D**). (**B**) Scatter plot with a fitting line showing the relation between coherence and PANSS8P1 (delusion). (**C**) Scatter plot with a fitting line showing the relation between coherence and PANSS8G9 (unusual thought content). (**D**) Scatter plot with a fitting line showing the relation between coherence and TLI global scores for disorganization in thought. The coherence ratings are jittered only for visualization to prevent overlapping points and make the distribution clearer, without affecting the actual data values in statistical analyses.

For assessing coherence in clinical speech, we analyzed the picture descriptions from ninety-four native English speakers. Subjects were asked to describe three pictures from the Thematic Apperception Test^40^ (one minute for each picture). These subjects are identical to what is reported in He et al.^10^; demographic and clinical data are available in Table 1. They were categorized as healthy controls (HC, *n* = 29), clinical high-risk (CHR, *n* = 18), first-episode psychosis (FEP, *n* = 29), and chronic schizophrenia (CS, *n* = 18). Human coherence ratings by three independent initial blind raters and a fourth consensus rating were averaged across the pictures and thresholded with the same criteria to produce a three-level ordinal label (low ≤ 1.8 < medium ≤ 2.2 < high)^36^. As shown in Fig. 3A, an ordered probit model, controlling for age, sex, and education, revealed a significant decline in discourse coherence in the FEP group (*z* = -4.567, *p* < 0.001), a mitigated yet significant decline in the CS group (*z* = -2.183, *p* = 0.029), and insignificant decline in the CHR group (*z* = -1.132, *p* = 0.258).

**Table 1:** Demographic characteristics and clinical assessment of the subjects.

	HC	CHR	FEP	CS	Test	Statistics	*p*
Number	29	18	29	18	–	–	–
Age	22.00 (3.00)	21.00 (5.75)	22.00 (4.00)	27.00 (6.50)	Kruskal-Wallis	19.362	0.000***
Sex	24.14%	22.22%	27.59%	27.78%	Pearson’s χ^2^ test	0.245	0.970
Education	68.97%	38.89%	51.72%	38.89%	Pearson’s χ^2^ test	6.231	0.101
SES (parental)	3.0 (2.0)	4.0 (0.0)	4.0 (3.0)	4.0 (2.0)	Kendall’s correlation	0.079	0.402
PANSS Missing	–	–	0	2	–	–	–
P1: Delusions	–	–	5.00 (2.00)	2.50 (3.00)	Kruskal-Wallis	58.095	0.000***
P2: Conceptual disorganization	–	–	3.00 (3.00)	1.00 (1.00)	Kruskal-Wallis	44.655	0.000***
P3: Hallucinatory behavior	–	–	5.00 (1.00)	3.00 (3.25)	Kruskal-Wallis	44.534	0.000***
N1: Blunted affect	–	–	2.00 (3.00)	1.00 (1.00)	Kruskal-Wallis	19.719	0.000***
N4: Passive/apathetic social withdrawal	–	–	3.00 (4.00)	1.00 (1.00)	Kruskal-Wallis	27.929	0.000***
N6: Lack of spontaneity and flow of conversation	–	–	1.00 (2.00)	1.00 ( 0.00)	Kruskal-Wallis	17.560	0.000***
G5: Mannerisms & posturing	–	–	1.00 (2.00)	1.00 ( 0.25)	Kruskal-Wallis	14.860	0.001***
G9: Unusual thought content	–	–	4.00 (2.00)	1.50 (1.25)	Kruskal-Wallis	44.792	0.000***
SOPS missing	–	2	–	–	–	–	–
SOPS Total	–	7.50 (6.00)	–	–	–	–	–
PANSS Positive	–	–	12.00 (4.00)	7.50 (5.25)	Kruskal-Wallis	61.398	0.000***
PANSS Negative	–	–	7.00 (6.00)	3.50 (1.75)	Kruskal-Wallis	32.979	0.000***
PANSS General	–	–	5.00 (4.00)	3.00 (2.00)	Kruskal-Wallis	44.972	0.000***
PANSS TOTAL	–	–	25.00 (9.00)	14.50 (9.50)	Kruskal-Wallis	58.585	0.000***
TLI Missing	2	0	0	1	–	–	–
Impoverishment of thought	0.00 ( 0.25)	0.50 ( 0.69)	0.25 ( 0.75)	0.25 ( 0.75)	Kruskal-Wallis	15.160	0.002**
Disorganization in thought	0.00 ( 0.25)	0.62 ( 0.69)	1.00 (1.25)	0.00 ( 0.50)	Kruskal-Wallis	18.437	0.000***

Cited from Table 1 in He et al.^10^. * *p* < 0.05, ** *p* < 0.01, *** *p* < 0.001. Age was indicated by the median (Interquartile range, IQR). There was 1 missing value of age in CS, which was fulfilled with the mean age of the CS group. Missing values in other variables were excluded from the analysis. Sex was represented by the percentage of female subjects. Education was indicated by the percentage of subjects with over 12 years of education. Socioeconomic status (SES), PANSS scores, and TLI scores were represented by the median (IQR). PANSS scores were only evaluated on the FEP and CS subjects. Symptom severity of the clinical high risk (CHR) participants was indicated by the total score of Scale of Prodromal Symptoms (SOPS). All four groups were assessed with Thought and Language Index (TLI).

Within FEP and CS subjects, we examined the relationship between coherence ratings and PANSS (Positive and Negative Syndrome Scale-8 items version), as well as the two global Thought Language Index (TLI) scores (respectively for impoverished and disorganized thought), using Spearman’s partial correlation, controlling for demographic covariates. The *p* values were corrected family-wise using FDR. As shown in Fig. 3, significant correlations were observed between coherence and the PANSS items delusions (*r* = -0.424, *q* = 0.015) and unusual thought content (*r* = -0.368, *q* = 0.033), and with TLI disorganization in thought (*r* = -0.392, *q* = 0.020).

### Semantic and probabilistic correlates of (in)coherence in SSD

The six variables reported above as “passing” the previous evaluation in the crowdsourced datasets were entered into the following analyses, in addition to two averaged semantic similarity scores due to their special interest from previous studies of coherence in SSD. We applied a two-step scheme for investigating the effects of these eight measures in SSD, using similar ordered probit models controlling for demographics and group differences. Firstly, we built one model for every feature to observe the main effects of semantic and probabilistic measures on coherence. Significant effects were observed only in three: word-level perplexity (Word_PPL, *z* = -1.983, *p* = 0.047), averaged semantic similarity with BERT (BERT_MeanK1, *z* = 2.066, *p* = 0.039), and skewness of sentence similarity distribution (Sent_Skew, *z* = -2.874, *p* = 0.004). The signs of *z* values agreed with the direction of correlations in the GCDC dataset.

**Fig. 4:**
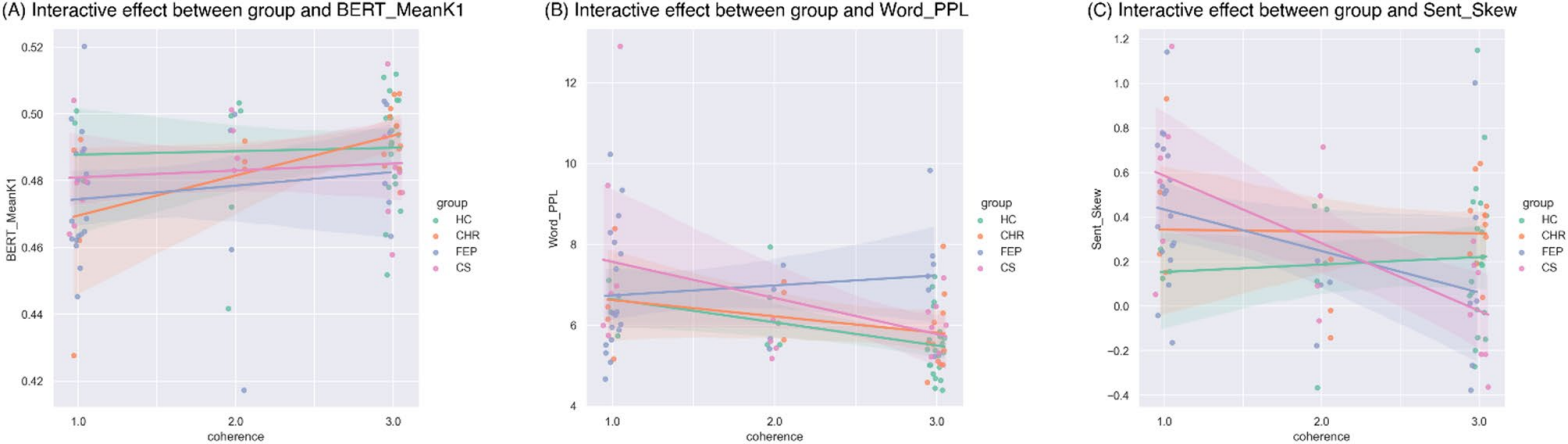
Scatter plot grouped by diagnosis with fitting lines indicating interaction effects on coherence between diagnosis group and (**A**) averaged semantic similarity between consecutive tokens from BERT models (BERT_MeanK1), (**B**) perplexity based on next token prediction probability from Mistral models (Word_PPL), (**C**) skewness of the distribution of sentence similarity scores (Sent_Skew). Crossing lines indicated possible interactive effects. The coherence ratings are jittered only for visualization to prevent overlapping points and make the distribution clearer, without affecting the actual data values in statistical analyses.

Then, as significant group effects were observed, we included group and each of the three measures, as well as their interactions, in the models. As shown in Fig. 4A, no significant interactional effects were observed between group and BERT_MeanK1. However, as shown in Fig. 4B, speech incoherence in FEP was moderated by word-level perplexity (*z* = 2.330, *p* = 0.020), with higher perplexity indicating more incoherent speech. No significant interactional effects were observed for word-level perplexity with CHR or CS. As shown in Fig. 4C, speech incoherence in CS was moderated by skewness of sentence similarity distribution (*z* = -2.287, *p* = 0.022), with more positively skewed sentence similarity distribution indicating more incoherence.

### Neural correlates of incoherence in SSD

**Fig. 5:**
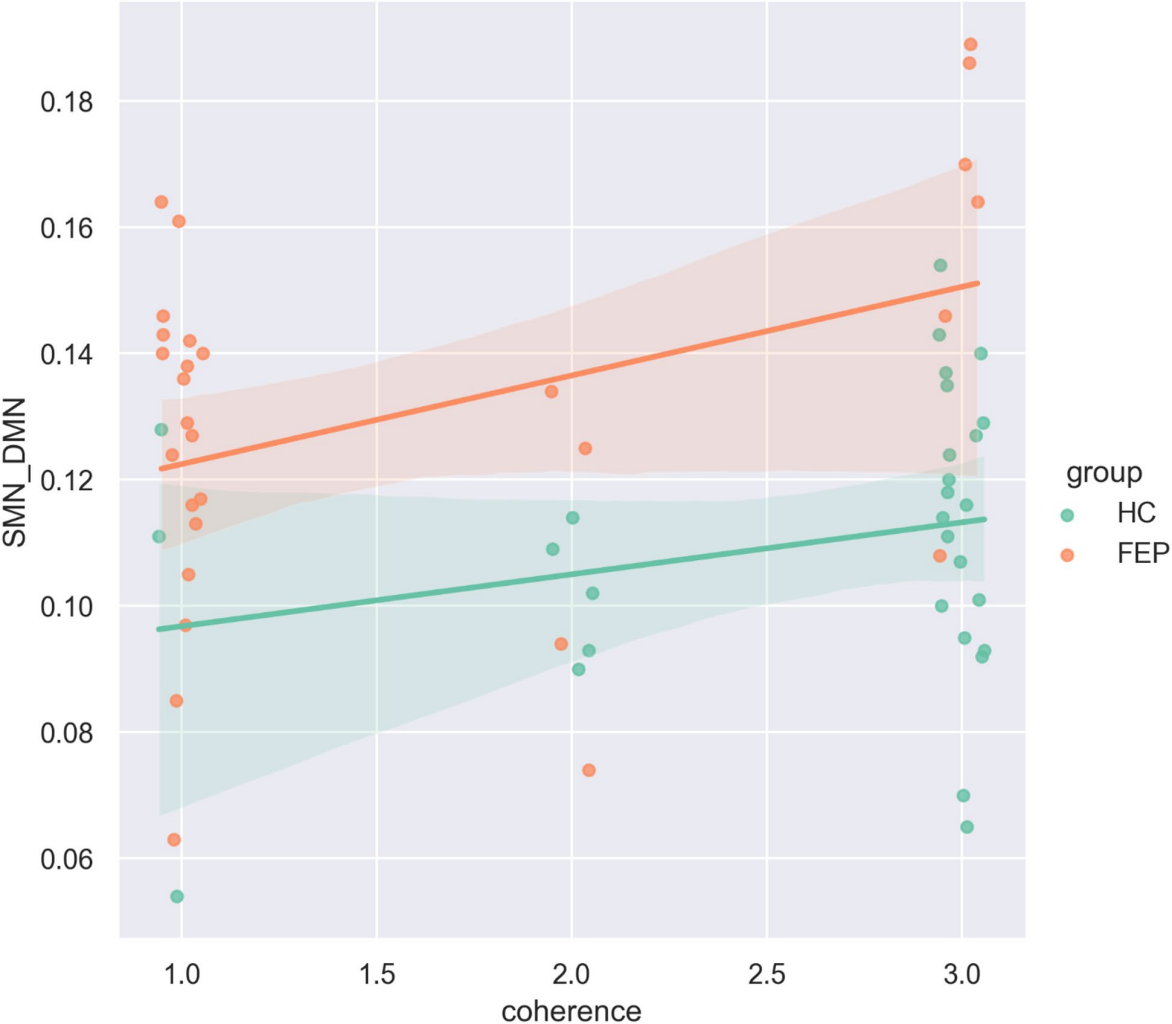
Scatter plot grouped by diagnosis with fitting lines indicating interaction effects on coherence between diagnosis group and gradient dispersion between the somatomotor network (SMN) and the default mode network (DMN). The coherence ratings are jittered only for visualization to prevent overlapping points and make the distribution clearer, without affecting the actual data values in statistical analyses.

A subset of the SSD dataset, comprising the 29 HC and 29 FEP, underwent resting-state fMRI scans on a Siemens 7T Plus (Erlangen, Germany) immediately after the picture descriptions. As noted in the introduction, a previous study using the same dataset reported that the gradient dispersion between VN and DMN as compressed in FEP while the gradient dispersion between SMN and DMN inflated^24^. In turn, within a semantic network of left default mode and limbic regions, the principal gradient revealed a hierarchical organization within this network, which was again compressed in FEP^24^. Similarly to the semantic correlation analysis, we first constructed a main effect model for each of the three dispersion scores, between VN and DMN, between SMN and DMN, and within the principal semantic network gradient. As shown in Fig. 5, greater dispersion between SMN and DMN were related to higher coherence (*z* = 2.536, *p* = 0.011). An additional model including the interaction effect revealed no significant interaction with group differences in coherence (*z* = -0.082, *p* = 0.934).

## Discussion

Coherence is a key notion in need of quantification when studying mental disorders in a linguistic perspective. This study sought to provide foundational evidence bearing on whether a broad range of currently prominent computational semantic measures can approximate this phenomenon as humanly perceived and rated, in health and disease; that this approximation is generalizable across languages; and that a loss of coherence can be an overt reflection of a significant change in brain-functional organization. Our results confirm the clinical significance of this aim: patients in a first acute psychotic state were indeed less coherent when describing pictures, and such human-rated incoherence correlated with delusions, unusual thought content, and disorganized thought. Yet, relations of the computational measures to human-rated coherence in neurotypical texts were scant, with only 6 out of 131 measures ‘passing’ the test of generalizability across three languages, with only weak correlations seen. None of these 6 measures included word-level semantic similarity metrics, while including sentence-based ones when approached graph-theoretically and with the BERT model, as well as probabilistic ones (next-word/sentence prediction). Notably, however, correlations remained weak for the latter as well.

The primary inference here, thus, is comparative. Among a broad set of candidate proxies, measures based on word-level semantic similarity from non-contextual embeddings showed no robust association with coherence across languages, whereas probabilistic predictability measures, most notably word-level perplexity, showed the most consistent alignment with human ratings. This first set of results reinforces the idea that coherence is a global phenomenon that is difficult to capture computationally through local (word-to-word) semantic relationships, particularly as measured with non-contextual models such as fastText, which fail to capture the grammatical context. Even at a sentential level, the weakness of the correlations and unclear reasons for the selectivity of the correlating measures urge caution on continuing to interpret these measures as targeting coherence, as has been done relatively routinely in the field^3–7^. It is unlikely that any single automated semantic metric can serve as a stand-alone substitute for clinical judgment. Rather, progress will require new computational measures that better integrate information across linguistic levels and contextual scales to capture discourse coherence as a complex, emergent phenomenon.

Notably, probabilistic measures carried clinically informative signal beyond their cross-sectional correlation with ratings. Specifically, perplexity moderated speech incoherence in FEP, such that higher perplexity was associated with more incoherent speech. Since model-based perplexity reflects the model’s prediction of the next word based on information in the preceding context, it cannot be regarded as a ‘semantic’ measure in any narrow sense. It rather reflects the integration of linguistic information at all levels, to get predictions of the next word right. It is thus of considerable interest to ask which layers of linguistic organization contribute what to these predictions. Interestingly, a recent study of Sharpe et al.^26^ shows that an expected effect, namely of predictions improving as the contextual window widens, *decreases* in SSD, and that this relative insensitivity to global context related to individual positive thought disorder scores. Given that a ‘context’ goes beyond a sequence of words, but is constructed through grammar giving rise to hierarchical structures, this raises more specific questions such as whether grammatical structure in the relevant context window would influence model predictions in SSD in a different way than in controls. If so, this would suggest a loosening of the effect that grammar exerts on how we transition from one word to the next, when building coherence.

In the same perspective, neurocognitive questions arise. Our results make it unlikely that any of our measures would target a specific mechanism that provides an underlying neurocognitive substrate of coherence. More likely, coherence is a global and emergent phenomenon arising from the integration of multiple factors and mechanisms. An integration across neurocognitive subsystems is approximated through whole-cortical gradients of functional connectivity. Individual variation in these has previously already been shown to relate to individual variation in language function and the control of meaning in particular^41^. Consistent with this perspective, we found that the dispersion of the principal gradient – featuring primary somatomotor and visual cortices at one end and the multi- or transmodal DMN at the other – positively correlates with human-rated coherence. This would be expected if coherence is not a word-to-word affair, but the result of integrating what we perceive with how we think, at a verbal level, and hence a global phenomenon. Strong overlap between the semantic network and the DMN has long been noted^42,43^; and a relation between compression of cortical gradients and computational semantic measures has been found^24^. This reinforces the perspective that an essentially freely produced and widely available human action – speech – could function as a primary readout of neurofunctional changes, increasing the need for identifying principles of brain function that could play an explanatory role for clinical speech deviations.

This work has several limitations. The first limitation is the assumption that cross-lingual linguistic patterns for coherence construction exist, which led us to focus on generalizable patterns rather than language-specific ones. This choice was motivated by the well-established, language-agnostic nature of coherence loss in SSD, as well as repeated findings of a ‘shrinking semantic space’ in SSD across various languages^9–12^. Another limitation arises from the use of models from the same family, which, while sharing similar architectures, could still introduce confounds due to model differences. We opted for this approach rather than using multilingual models, as monolingual models typically outperform multilingual ones in non-English languages (e.g.^44^). Additionally, we acknowledge the heterogeneity of genres within the neurotypical datasets and the differences between neurotypical written texts and spontaneous speech in patients. Differences between written and spoken discourse (e.g., planning demands, disfluencies, and turn-level structuring), between normative and clinical language, as well as between written genres and oral picture descriptions, may attenuate alignment with human ratings and therefore limit the direct generalizability of the observed correlations. While we lack homogeneous datasets to address this limitation directly, these variations, on the other hand, may enhance the robustness of the generalizable patterns observed. Furthermore, the conceptual motivation for coherence disturbances in schizophrenia stems from formal thought disorder (loosening of associations), which is not specific to written versus spoken production. Indeed, prior work has reported increased consecutive semantic similarity in both modalities^10,45^. We need to expect correlation patterns that can generalize across different genres and tasks. Also, medication status differed within the CSZ group and between CSZ and FEP, and medication can affect speech production. More broadly, the relatively small sample size serves as one limitation and the observational nature of the study limit our ability to make causal claims.

In summary, our findings validated the incoherent nature of speech in schizophrenia patients and showed its symptomatic dimensions, but provided reasons to question the widely held assumption of semantic space measures, especially semantic similarity among words, serving as incoherence markers. We anticipate that future studies will move beyond the semantic space measures to develop metrics representing how different linguistic levels integrate into a discourse and shed light on how coherence relates to such integration.

## Materials and methods

### Data

In this study, we employed three coherence datasets from the general population and one clinical dataset. The three coherence datasets, GCDC in English, CEDCC in Chinese, and DDisCo in Danish, are available publicly. The clinical dataset (clinical high-risk (CHR, *n* = 18), first-episode psychosis (FEP, *n* = 29), chronic schizophrenia (CS, *n* = 18), and healthy controls (HC, *n* = 29)), is identical to the data used in our other reports^10,24^. For details, please see there. In brief, the majority of FEP subjects were assessed during the first week after referral to the first-episode unit and had < 2 weeks of lifetime antipsychotic exposure. Only those subjects with a confirmed diagnosis of schizophrenia after 6 months were kept in this study. CHR subjects featured subthreshold psychosis (Attenuated Psychosis Syndrome or brief and limited intermittent psychosis (BLIPS)) as per the Brief Structured Interview for Psychosis-risk Syndromes (SIPS) and had no prior exposure to antipsychotics. CS subjects were clinically stable with > 3 years after illness onset. They had no recorded hospitalization in the past year and received community-based care from physicians affiliated to a first-episode clinic (PEPP, London Ontario). All patients provided written informed consent as stipulated by the Research Ethics Committee of University of Western Ontario, London, Canada (ID 108268).

### Text processing

We defined three different levels of meaningful units in a text: lexical categories, subwords, and sentences. Texts were split into sentences and words with part-of-speech tags using spaCy models (version 3.7.4, English model: en_core_web_lg, Chinese model: zh_core_web_lg, and Danish model: da_core_news_lg). The psychotic speech was split into utterances manually and every utterance was tokenized using the identical English model from spaCy. Lexical categories were defined as nouns, verbs, and adjectives, and encoded with the fastText model pretrained on the corresponding languages^46^. Secondly, the texts were tokenized into subwords and encoded into contextual embeddings by BERT models for each language (English: bert-base-uncased; Chinese: hfl/chinese-macbert-large; Danish: Maltehb/danish-bert-botxo). Finally, the meanings of sentences (or utterances in the psychotic speech samples) were represented by sentential meaning models (English: Alibaba-NLP/gte-large-en-v1.5; Chinese: shibing624/text2vec-base-chinese; Danish: Alibaba-NLP/gte-multilingual-base). In addition, we introduced Mistral generative models for probability-based metrics (English: mistralai/Mistral-7B-v0.1; Chinese: itpossible/Chinese-Mistral-7B-v0.1; Danish: danish-foundation-models/munin-7b-alpha). All models used in this study are publicly available from Hugging Face, with details in supplementary table S1.

### Feature extraction

A comprehensive set of features was developed to explore the power of semantic similarity measures in predicting coherence based on the previous work in schizophrenic speech and semantic space. As explained above, we started from averaged semantic similarity and included more measures describing similarity distribution, temporal dynamics, similarity to the centroid as a proxy for the topic, graph-theoretic properties, and predictability. Formulas for these measures could be found in SI. For every measure, only text with over four units entered the semantic analysis, while texts with less than four units were treated as missing values.

#### Averaged semantic similarity

Our comprehensive set of semantic measures started from the most commonly used measure, the averaged semantic similarity between consecutive meaningful units (first-order mean – MeanK1). Then, we computed the cosine similarity between every pair of consecutive meaningful units with one unit in between, that is, the second order semantic similarity (MeanK2), and the global averaged cosine similarity between all binary pairs of meaningful units, either consecutive or not (Global). These mean scores, either consecutive at different orders or global ones, have often been found as indicative of speech in psychosis in previous work with the name of coherence^13,47,48^.

#### Statistical distribution descriptors

To extract more information from the semantic similarity measure, we then treated the semantic similarity between consecutive units as an array with temporal order, from the first binary pair to the last binary pairs. Six statistical descriptors were introduced to characterize data distribution similarities, including variance (Var), maximum (Peak), minimum (Valley), amplitude (Amp, difference between maximum and minimum), skewness (Skew), and excess kurtosis (Kurt, i.e. kurtosis – 3, where 3 is the kurtosis of a normal distribution).

#### Temporal dynamics of semantic similarity waves

As discourse coherence is established along with its temporal development, we took the temporal order of the sequence into account as a wave function, and depicted the temporal dynamics of these semantic similarity waves, through the mean-crossing rate (MCR) of the wave function, its number of slope sign changes (SSC), the wave length (WL), its approximate entropy (ApEn), its autocorrelation coefficient at one lag (ACF), and the zero-crossing rate of the ACF waveform at different number of lags (AcfZcr).

#### Graph-theoretical properties

Moreover, we represented a text as a small-world graph, with meaningful units as nodes and thresholded semantic similarity as edges. Graph-theoretical properties show how these meaningful units integrate into a whole graph (closeness centrality (CC)) with proper segregations (averaged clustering coefficients (Cluster)). The integration and segregation of meaningful units were previously found to get disrupted in schizophrenia^24^, which may relate to coherence.

#### Semantic centroid analysis

In addition to similarity among the units, the similarity between units and their centroids, a proxy for the topic, has been found to be indicative of coherence^49^. Thus, we defined two centroids: The static centroid (stat) was defined as the averaged embedding across all units, approximating the topic of the whole discourse; while the cumulative centroid (cuml) was defined as the averaged embedding over all preceding units, including the temporal perspective of topic changes along with discourse development. We computed the similarity between every unit and a centroid, whether static or cumulative, as a time series from the first unit to the last unit. Then we extracted the mean score, distributional descriptors, and dynamical measures as described above.

#### Probability-based metrics

Finally, we introduced two probability-based metrics. Word-level perplexity (Word_PPL) was defined as the exponential of the average negative log-likelihood of a sequence of words, using Mistral models^39^. The sentence-level perplexity (BERT_NSP_PPL) was defined as the exponential of the average negative log-likelihood of a sequence of sentences, based on the probability scores from next sentence prediction (NSP) by BERT models.

### Incoherence in SSD: group differences, clinical dimensions, semantic metrics, and neural correlates

Speech samples were rated by three experts, two with linguistic background and one with medical background, who were blinded to the diagnosis and clinical ratings before and during the ratings. The inter-rater reliability was comparable to that of the GCDC corpus, with intraclass correlation (ICC2k) of 0.573 and averaged weighted Cohen’s *κ* of 0.309, indicating merely a fair level of agreement. This is broadly consistent with the agreement observed in the expert ratings on the GCDC English dataset (weighted Cohen’s *κ* on the subsets: Yahoo = 0.386, Clinton = 0.250, Enron = 0.273, Yelp = 0.181)^36^. To address discrepancies among the three raters, we solicited a senior expert with extensive experience in language in schizophrenia to provide an adjudicated consensus rating based on the raters’ prior evaluations, using his expert judgment only to resolve ambiguous cases. As every subject yielded three speech samples, the coherence scores were averaged across the three samples and thresholded with the same criteria to produce a three-level ordinal label (low ≤ 1.8 < medium ≤ 2.2 < high)^36^.

FEP and CS subjects were assessed with the Positive and Negative Syndrome Scale-8 items version (PANSS), with delusions (PANSS8P1), conceptual disorganization (PANSS8P2), hallucinatory behavior (PANSS8P3), blunted affect (PANSS8N1), passive/apathetic social withdrawal (PANSS8N4), lack of spontaneity/flow of conversation (PANSS8N6), mannerisms/posturing (PANSS8G5), and unusual thought content (PANSS8G9)^50^. Elicited speech was scored using the Thought Language Index (TLI), with scores for impoverishment of thought (TLIGIOT) and for disorganization in thought (TLIGDIT)^51^.

As coherence is an ordinal variable, an ordered probit model was applied with coherence as the response variable, diagnosis group as one regressor (HC as the reference category), and age, sex, and education as covariates. The model was fit on the data using Broyden-Fletcher-Goldfarb-Shanno (BFGS) method. Fitting formula for the main effects:

coherence ~ age + C(Gender) + C(Education) + C(group, Treatment(“HC”)).

Then, we related the coherence ratings to eight PANSS items, as well as two global TLI scores, using Spearman’s partial correlation, with age, sex, and education as covariates.

Similarly, we constructed ordered probit models for relating semantic metrics to coherence. We first examined the main effect of semantic metrics, with coherence as the response variable, each of the semantic measures as one regressor, and age, sex, and education as covariates. Then, we added the diagnosis group, and the interaction between diagnosis group and each semantic measure into the model. Fitting formula for the main effects:

coherence ~ age + C(Gender) + C(Education) + linguistic_feature + C(group, Treatment(“HC”)).

Fitting formula for the interactional effects:

coherence ~ age + C(Gender) + C(Education) + linguistic_feature + C(group, Treatment(“HC”)) + linguistic_feature*C(group, Treatment(“HC”)).

### fMRI image acquisition and preprocessing

The fMRI data was acquired at the Centre for Functional and Metabolic Mapping (CFMM) at the University of Western Ontario on a Siemens 7 T Plus (Erlangen, Germany). A total of 360 whole-brain functional images were collected using a multi-band EPI acquisition sequence with 20 ms of echo time, 1000 ms of repetition time, a flip angle of 30◦, in 63 slices with a multi-band factor of 3, iPat of 3, and an isotropic resolution of 2 mm. The T1-weighted MP2RAGE anatomical volume was acquired at a 750 μm isotropic resolution (TE/TR = 2.83/6000 ms). Images were preprocessed using fMRIPrep 21.0.1. Details on how fMRIPrep preprocessed the anatomical and functional data can be found in the SI.

### Functional connectivity gradient analysis

The details of functional connectivity (FC) gradient analysis can be found in^24^. In summary, functional time series were extracted from Schaefer 1000-parcels^52^ as organized into seven Yeo networks^53^: visual network (VN), somatomotor network (SMN), dorsal attention (DAN), ventral attention (VAN), limbic (LN), frontoparietal control (FPN), and default mode network (DMN). For every subject, we generated a 1000 × 1000 FC matrix using z-transformed correlation coefficients of the time series. The group FC templates, one for HC and another for FEP, were averaged from the individual FC matrices. BrainSpace^54^ was employed to map the group FC templates to low-dimensional manifolds comprising 10 gradients. We also carried out this pipeline on every subject’s FC matrix. Subject-level gradient maps were aligned to the group-level gradients with generalized Procrustes rotation for better robustness. We focused on the first gradients, G1 and G2, due to their more established relationship with cognitive functions^55^, reflecting the separation between unimodal and transmodal cortex (i.e., from VN and SMN to DMN)^56^.

Such gradient analysis was applied also to a semantic network defined as comprising the left default and limbic regions, following Binder et al.^42^. Within the semantic network, we first computed the 10 gradients from FC matrices, analyzed the first gradient, and then defined the dispersion of this first gradient of the semantic network as the sum squared Euclidean distance of every node to its centroid.

Fitting formula for the main effects of gradient dispersion on linguistic coherence:

coherence ~ age + C(Gender) + C(Education) + gradient_dispersion + C(group, Treatment(“HC”)).

## Supplementary Information

Below is the link to the electronic supplementary material.

Supplementary Material 1

## Data Availability

Interrater reliability was analyzed with JASP 0.16.4.0. All other analyses were carried out with self-developed Python scripts (Python 3.11.7). Ordinal regression model was fit with statsmodels 0.14.0. Spearman’s correlation analyses were conducted with pingouin 0.5.4. Gradient analyses utilized brainspace 0.1.4 and brainstat 0.4.2. Visualization utilized matplotlib 3.10.1 and seaborn 0.13.2. Other packages involved: numpy 1.26.4, pandas 2.2.3, torch 2.0.1, spacy 3.7.4, transformers 4.48.3, fasttext 0.9.2, network 3.1, scipy 1.12.0, and antropy 0.1.9. All scripts and derived measures from the three datasets from the general population are available at [https://github.com/RuiHe1999/semantic_marker_coherence](https:/github.com/RuiHe1999/semantic_marker_coherence) . Datasets from the general population could be found via the original repository. Transcripts of the psychotic speech used for this study as well as anonymized clinical scores, are available from LP (write to lpalaniy@uwo.ca) upon reasonable request within the stipulations laid by The Research Ethics Committee of University of Western Ontario, London, Canada (Project ID: 108268; Most recent review reference: 2022-108268-71496; Study Title: The Pathophysiology of Thought Disorder in Psychosis (TOPSY)).
