# Supplementary material for: Reduced linguistic coherence in psychosis defies semantic similarity accounts and relates to altered large-scale cortical hierarchy
Source: Sci Rep. 2026 Feb 8;16:7799. doi: 10.1038/s41598-026-39025-1 (PMC12948966; doi:10.1038/s41598-026-39025-1)
Supplement: Supplementary file 1 — Supplementary Material 1 [file 41598_2026_39025_MOESM1_ESM.docx]

**Semantic measures**

Averaged semantic similarity

Given a text split into $N$ units $\{U_{1},U_{2},\ldots,U_{N}\}$, we vectorized each unit with a certain language model into a matrix of embeddings $\{e_{1},e_{2},\ldots,e_{N}\}$. The similarity between vectors $e_{i}$ and $e_{j}$ was defined by the cosine value of the angle between them:

$$\begin{aligned} {Sim}_{i,j}=\frac{e_{i}\cdot e_{j}}{|e_{i}||e_{j}|}\#\left( SEQ Equation \backslash* ARABIC 1 \right) \end{aligned}$$

where $|e_{i}|$ and $|e_{j}|$ are the norms of the vectors $e_{i}$ and $e_{j}$, respectively.

We first computed the averaged cosine similarity between adjacent words as first-order mean similarity (MeanK1):

$$\begin{aligned} MeanK1=\frac{1}{N-1}\sum_{i=1}^{N-1} Sim_{i,i+1}\#\left( SEQ Equation \backslash* ARABIC 2 \right) \end{aligned}$$

Then, the second-order mean similarity was defined as the averaged cosine similarity between two units with one unit in between (MeanK2):

$$\begin{aligned} MeanK2=\frac{1}{N-2}\sum_{i=1}^{N-2} Sim_{i,i+2}\#\left( SEQ Equation \backslash* ARABIC 3 \right) \end{aligned}$$

Global semantic similarity was defined as the averaged cosine similarity between all unit pairs:

$$\begin{aligned} Global=\frac{2}{N\left( N-1 \right)}\sum_{i=1}^{N-1} \sum_{j=i+1}^{N} Sim_{i,j}\#\left( SEQ Equation \backslash* ARABIC 4 \right) \end{aligned}$$

Temporal dynamics of semantic similarity score

Given a time series of semantic similarity between adjacent units $\{Sim_{1,2},Sim_{2,3},\ldots,Sim_{N-1,N}\}$, let $x_{i}$ represent the *i*th semantic similarity in the array $\{Sim_{1,2},Sim_{2,3},\ldots,Sim_{N-1,N}\}$ and $\bar{x}$represent the mean value of this array (i.e. MeanK1).

The mean crossing rate (MCR) measures how frequently a semantic similarity score crosses its mean value $\bar{x}$:

$$\begin{aligned} MCR=\frac{1}{N-1}\left( \sum_{i=1}^{N-1} \left[ \text{sign}\left( x_{i}-\bar{x} \right)\neq\text{sign}\left( x_{i+1}-\bar{x} \right) \right]-\left[ x_{i}=\bar{x} \right] \right)\#\left( SEQ Equation \backslash* ARABIC 5 \right) \end{aligned}$$

Slope sign changes (SSC) measures the normalized number of times the slope of the signal changes its sign:

$$\begin{aligned} SSC=\frac{1}{N-2}\sum_{i=2}^{N-1} \mathbb{1}\left( \left( x_{i}-x_{i-1} \right)\cdot\left( x_{i}-x_{i+1} \right)>0 \right)\#\left( SEQ Equation \backslash* ARABIC 6 \right) \end{aligned}$$

where $\mathbb{1}$ is an indicator function that returns 1 if the condition inside is true, and 0 otherwise.

The wave length (WL) calculates the average absolute difference between two consecutive semantic similarity scores:

$$\begin{aligned} WL=\frac{1}{N-1}\sum_{i=1}^{N-1} \left| x_{i+1}-x_{i} \right|\#\left( SEQ Equation \backslash* ARABIC 7 \right) \end{aligned}$$

Approximate entropy (ApEn) measures the unpredictability of fluctuations over time. Higher ApEn indicates a more predictable time series with an increasing amount of regularity in its fluctuations. ApEn was estimated using the Python package called *Antropy* with default parameters.

Autocorrelation function (ACF) computes the correlation coefficients between the time series and copies of itself that are temporally shifted with a series of lags:

$$\begin{aligned} \text{ACF}_{k}=\frac{\sum_{i=1}^{N-k} \left( x_{i}-\bar{x} \right)\left( x_{i+k}-\bar{x} \right)}{\sum_{i=1}^{N} \left( x_{i}-\bar{x} \right)^{2}}\#\left( SEQ Equation \backslash* ARABIC 8 \right) \end{aligned}$$

where *k* is the number of lags. We extracted ACF with one lag and zero crossing rate of the ACF waveform (AcfZcr).

Graph-theoretical properties

We computed the cosine similarity scores between every pair of two meaningful units for an affinity matrix binarized by proportional thresholding. Consistent with previous studies, we did not pick a single threshold in this case but selected the lowest threshold from 0.05 to 0.8 with intervals of 0.05, which returns a sparsified matrix with the average degree (the degree of a node is the number of connections linked to the node) over all nodes larger than two multiplies the e-base logarithm of the number of nodes ($2\log N$) (Zhang et al., 2011). This procedure assures that the thresholded network exhibits small-world properties (Watts and Strogatz, 1998) with as few edges as possible. Notably, we did not require the small-worldness coefficients to be larger than 1.1 as done in some previous studies, as this is a value derived from specific brain graph data and did not apply to the semantic graph. We used the *networkx* package for computing the closeness centrality and clustering coefficient.

Semantic centroid analysis

Given a matrix of embeddings $\{e_{1},e_{2},\ldots,e_{N}\}$, the static centroid was defined as the averaged embedding across all units:

$$\begin{aligned} C_{stat}=\frac{1}{N}\sum_{i=1}^{N} e_{i}\#\left( SEQ Equation \backslash* ARABIC 9 \right) \end{aligned}$$

The cumulative centroid was defined as the averaged embedding over all preceding units. The cumulative centroid at the *j*th unit is thus (*j* > 1):

$$\begin{aligned} C_{cuml,j}=\frac{1}{j-1}\sum_{i=1}^{j-1} e_{i}\#\left( SEQ Equation \backslash* ARABIC 10 \right) \end{aligned}$$

Semantic perplexity analysis

Given a text split into $N$ sentences $\{S_{1},S_{2},\ldots,S_{N}\}$, the probability of the sentence $S_{i}$ was retrieved from BERT models as the probability of being the next sentence of $S_{i-1}$ :

$$\begin{aligned} \boldsymbol{P}\left( \boldsymbol{S}_{\boldsymbol{i}} \right)\boldsymbol{=P}\left( \boldsymbol{S}_{\boldsymbol{i}} | \boldsymbol{S}_{\boldsymbol{i}}\boldsymbol{-1} \right)\boldsymbol{\#}\left( SEQ Equation \backslash* ARABIC 11 \right) \end{aligned}$$

Given a text split into $N$ tokens $\{T_{1},T_{2},\ldots,T_{N}\}$, the probability of the token $T_{i}$ was retrieved from Mistral models as the probability being the next tokens conditioned on all preceding contexts $T_{<i}$:

$$\begin{aligned} \boldsymbol{P}\left( \boldsymbol{T}_{\boldsymbol{i}} \right)\boldsymbol{=P}\left( \boldsymbol{T}_{\boldsymbol{i}} | \boldsymbol{T}_{\boldsymbol{<i}} \right)\boldsymbol{\#}\left( SEQ Equation \backslash* ARABIC 12 \right) \end{aligned}$$

Given a text split into $N$ units $\{U_{1},U_{2},\ldots,U_{N}\}$, let the units be either sentences or tokens, the perplexity (PPL) of the text is defined as the exponential values of averaged log-likelihood:

$$\begin{aligned} PPL=\exp(\frac{1}{N}\sum_{i=2}^{N} \log P\left( U_{i} \right))\#\left( SEQ Equation \backslash* ARABIC 13 \right) \end{aligned}$$

**Semantic correlates of coherence in neurotypical language**

As shown in Fig. S1, there were 28 measures evaluated as uncertain, most of which, except the BERT_CC were significant in the English data. From these “uncertain” measures, lower coherence might correlate with:

- Higher consecutive averaged semantic similarity between two lexical categories with one lexical category in between
- Lower consecutive averaged semantic similarity between two sentences with one sentence in between
- Lower global averaged semantic similarity between all binary pairs of sentences
- More variance in the distribution of lexical category similarity scores
- Lower mean-crossing rate in the wave of lexical category similarity scores
- Less sign slope changes in the wave of lexical category similarity scores
- Higher maximum semantic similarity between two BERT tokens
- Higher excess kurtosis in the distribution of BERT token similarity scores
- Lower maximum semantic similarity between two sentences
- Lower minimum semantic similarity between two sentences
- Higher approximate entropy in the wave of sentence similarity scores (i.e. less predictable)
- Higher closeness centrality of BERT-based graph
- Lower closeness centrality of sentence-based graph
- Less clusters in the sentence-based graph
- More variance in the similarity between lexical categories and their static centroid
- Larger differences between the maximum and minimum scores in the similarity between lexical categories and their static centroid
- Less variance in the similarity between BERT tokens and their cumulative centroid
- Shorter wave length in the wave of similarity between BERT tokens and their cumulative centroid
- Further deviation from the static sentence centroid
- Lower maximum semantic similarity between sentences and their static centroid
- Lower minimum semantic similarity between sentences and their static centroid
- Higher skewness in the distribution of similarity between sentences and their static centroid
- Lower maximum semantic similarity between sentences and their cumulative centroid
- Lower minimum semantic similarity between sentences and their cumulative centroid
- Larger differences between the maximum and minimum scores in the similarity between sentences and their cumulative centroid
- Higher excess kurtosis in the distribution of similarity between sentences and their cumulative centroid
- Higher approximate entropy in the wave of similarity between sentences and their cumulative centroid (i.e. less predictable)
- Higher autocorrelation coefficients in the wave of similarity between sentences and their cumulative centroid

Summarize the findings conceptually. Results indicated distinct patterns between the sentence-based models and word-based models in their relations to coherence. To retain a high level of coherence, at the word level, both lexical categories and BERT tokens need to be *un*similar to each other (i.e. an inflating semantic space), as contradictory to the traditional belief. The distribution of lexical category similarities needs to retain at a stable level (i.e. less variance in total), but fluctuate around this stable level more in the temporal course of discourse development. They also need to stay stable around their static centroid.

To be highly coherence, the distribution of BERT token similarities needs to distribute more normally and more concentrated about the mean than the platykurtic distribution. BERT tokens need to be less stable around their cumulative centroid.

More sentence-level variables emerged here. Setences need to be similar to each other (i.e. a shrinking semantic space), aligning with the traditional belief, but this coexists with the findings that similarity also increases in the psychotic population who produced significantly more incoherent speech. The similarity among adjacent sentences, as well as that between sentences and their cumulative centroids, needs to have lower approximate entropy but also lower autocorrelation coefficients with one lag. In other words, the wave may need to be highly predictable as a whole but not very predictable in the short terms (e.g. one lag). This could happen in the case of simple periodic patterns which take relatively long time in one period. Sentences also need to form more clusters for coherence.

To be noted, these findings must be taken into account with the understanding that we could only observe WEAK correlations in two datasets.

Results on the whole feature set are reported in Fig. S2, Fig. S3, Fig. S4, Fig. S5, and Fig. S6.

**Information on reprocessing using fMRIPrep**

**Anatomical data preprocessing.** A total of 1 T1-weighted (T1w) images were found within the input BIDS dataset. The T1-weighted (T1w) image was corrected for intensity non-uniformity (INU) with N4BiasFieldCorrection (Tustison et al., 2010), distributed with ANTs 2.3.3 (Avants, Epstein, Grossman, & Gee, 2008), and used as T1w-reference throughout the workflow. The T1w-reference was then skull-stripped with a Nipype implementation of the antsBrainExtraction.sh workflow (from ANTs), using OASIS30ANTs as target template. Brain tissue segmentation of cerebrospinal fluid (CSF), white-matter (WM) and gray-matter (GM) was performed on the brain-extracted T1w using fast (FSL 6.0.5.1:57b01774, RRID:SCR_002823) (Zhang, Brady, & Smith, 2001). Brain surfaces were reconstructed using recon-all (FreeSurfer 6.0.1, RRID:SCR_001847) (Dale, Fischl, & Sereno, 1999), and the brain mask estimated previously was refined with a custom variation of the method to reconcile ANTs-derived and FreeSurfer-derived segmentations of the cortical gray-matter of Mindboggle (RRID:SCR_002438) (Klein et al., 2017). Volume-based spatial normalization to one standard space (MNI152NLin2009cAsym) was performed through nonlinear registration with antsRegistration (ANTs 2.3.3), using brain-extracted versions of both T1w reference and the T1w template. The following template was selected for spatial normalization: ICBM 152 Nonlinear Asymmetrical template version 2009c [RRID:SCR_008796; TemplateFlow ID: MNI152NLin2009cAsym] (Fonov, Evans, McKinstry, Almli, & Collins, 2009).

**Functional data preprocessing.** For each of the 1 BOLD runs found per subject (across all tasks and sessions), the following preprocessing was performed. First, a reference volume and its skull-stripped version were generated using a custom methodology of fMRIPrep. Head-motion parameters with respect to the BOLD reference (transformation matrices, and six corresponding rotation and translation parameters) are estimated before any spatiotemporal filtering using mcflirt (FSL 6.0.5.1:57b01774) (Jenkinson, Bannister, Brady, & Smith, 2002). The BOLD time-series (including slice-timing correction when applied) were resampled onto their original, native space by applying the transforms to correct for head-motion. These resampled BOLD time-series will be referred to as preprocessed BOLD in original space, or just preprocessed BOLD. The BOLD reference was then co-registered to the T1w reference using bbregister (FreeSurfer) which implements boundary-based registration (Greve & Fischl, 2009). Co-registration was configured with six degrees of freedom. Several confounding time-series were calculated based on the preprocessed BOLD: framewise displacement (FD), DVARS and three region-wise global signals. FD was computed using two formulations following Power (absolute sum of relative motions) (Power et al., 2014) and Jenkinson (relative root mean square displacement between affines) (Jenkinson et al., 2002). FD and DVARS are calculated for each functional run, both using their implementations in Nipype (following the definitions by Power et al. (2014)). The three global signals are extracted within the CSF, the WM, and the whole-brain masks. Additionally, a set of physiological regressors were extracted to allow for component-based noise correction (CompCor) (Behzadi, Restom, Liau, & Liu, 2007). Principal components are estimated after high-pass filtering the preprocessed BOLD time-series (using a discrete cosine filter with 128s cut-off) for the two CompCor variants: temporal (tCompCor) and anatomical (aCompCor). tCompCor components are then calculated from the top 2% variable voxels within the brain mask. For aCompCor, three probabilistic masks (CSF, WM and combined CSF+WM) are generated in anatomical space. The implementation differs from that of Behzadi et al. (2007) in that instead of eroding the masks by 2 pixels on BOLD space, the aCompCor masks are subtracted a mask of pixels that likely contain a volume fraction of GM. This mask is obtained by dilating a GM mask extracted from the FreeSurfer’s aseg segmentation, and it ensures components are not extracted from voxels containing a minimal fraction of GM. Finally, these masks are resampled into BOLD space and binarized by thresholding at 0.99 (as in the original implementation). Components are also calculated separately within the WM and CSF masks. For each CompCor decomposition, the k components with the largest singular values are retained, such that the retained components’ time series are sufficient to explain 50 percent of variance across the nuisance mask (CSF, WM, combined, or temporal). The remaining components are dropped from consideration. The head-motion estimates calculated in the correction step were also placed within the corresponding confounds file. The confound time series derived from head motion estimates and global signals were expanded with the inclusion of temporal derivatives and quadratic terms for each (Satterthwaite et al., 2013). Frames that exceeded a threshold of 0.5 mm FD or 1.5 standardised DVARS were annotated as motion outliers. The BOLD time-series were resampled into standard space, generating a preprocessed BOLD run in MNI152NLin2009cAsym space. First, a reference volume and its skull-stripped version were generated using a custom methodology of fMRIPrep. All resamplings can be performed with a single interpolation step by composing all the pertinent transformations (i.e. head-motion transform matrices, susceptibility distortion correction when available, and co-registrations to anatomical and output spaces). Gridded (volumetric) resamplings were performed using antsApplyTransforms (ANTs), configured with Lanczos interpolation to minimize the smoothing effects of other kernels (Lanczos, 1964). Non-gridded (surface) resamplings were performed using mri_vol2surf (FreeSurfer).

**Supplementary tables**

**Table S1.** Model details.

| Model name | Commit hash | Fine-tuned? | References |
| --- | --- | --- | --- |
| bert-base-uncased | 86b5e0934494bd15c9632b12f734a8a67f723594 | No | Devlin, J., Chang, M.-W., Lee, K., & Toutanova, K. (2018). BERT: Pre-training of deep bidirectional transformers for language understanding (arXiv:1810.04805). arXiv. |
| hfl/chinese-macbert-large | 1cf2677c782975600ce58e2961656b1b29eddbae | No | Cui, Y., Che, W., Liu, T., Qin, B., Wang, S., & Hu, G. (2020, November). *Revisiting pre-trained models for Chinese natural language processing*. In *Proceedings of the 2020 Conference on Empirical Methods in Natural Language Processing: Findings* (pp. 657–668). Association for Computational Linguistics. |
| Maltehb/danish-bert-botxo | aeffe01ee981726b623f33ae778187328222ec97 | No | https://github.com/certainlyio/nordic_bert |
| Alibaba-NLP/gte-large-en-v1.5 | 104333d6af6f97649377c2afbde10a7704870c7b | No | Zhang, X., Zhang, Y., Long, D., Xie, W., Dai, Z., Tang, J., Lin, H., Yang, B., Xie, P., Huang, F., et al. (2024). *mGTE: Generalized long-context text representation and reranking models for multilingual text retrieval* (arXiv:2407.19669). arXiv.  Li, Z., Zhang, X., Zhang, Y., Long, D., Xie, P., & Zhang, M. (2023). *Towards general text embeddings with multi-stage contrastive learning* (arXiv:2308.03281). arXiv. |
| shibing624/text2vec-base-chinese | 183bb99aa7af74355fb58d16edf8c13ae7c5433e | No | https://github.com/shibing624/text2vec |
| Alibaba-NLP/gte-multilingual-base | 9bbca17d9273fd0d03d5725c7a4b0f6b45142062 | No | Zhang, X., Zhang, Y., Long, D., Xie, W., Dai, Z., Tang, J., Lin, H., Yang, B., Xie, P., Huang, F., et al. (2024). *mGTE: Generalized long-context text representation and reranking models for multilingual text retrieval* (arXiv:2407.19669). arXiv.  Li, Z., Zhang, X., Zhang, Y., Long, D., Xie, P., & Zhang, M. (2023). *Towards general text embeddings with multi-stage contrastive learning* (arXiv:2308.03281). arXiv. |
| mistralai/Mistral-7B-v0.1 | 27d67f1b5f57dc0953326b2601d68371d40ea8da | No | Jiang, A. Q., Sablayrolles, A., Mensch, A., Bamford, C., Chaplot, D. S., de las Casas, D., Bressand, F., Lengyel, G., Lample, G., Saulnier, L., Renard Lavaud, L., Lachaux, M.-A., Stock, P., Le Scao, T., Lavril, T., Wang, T., Lacroix, T., & El Sayed, W. (2023). *Mistral 7B* (arXiv:2310.06825). arXiv. |
| itpossible/Chinese-Mistral-7B-v0.1 | 4151bd713447ceab74fafa27fe42c91262913066 | No | Chen, Z., Lin, M., Wang, Z., Zang, M., & Bai, Y. (2024). PreparedLLM: Effective pre-pretraining framework for domain-specific large language models. *Big Earth Data*, 1–24. <https://doi.org/10.1080/20964471.2024.2396159> |
| danish-foundation-models/munin-7b-alpha | 52dfaa51e4a19c3215e61b3ea6b4dc2150dadf10 | No | <https://github.com/danish-foundation-models/site> |

**Supplementary figures**


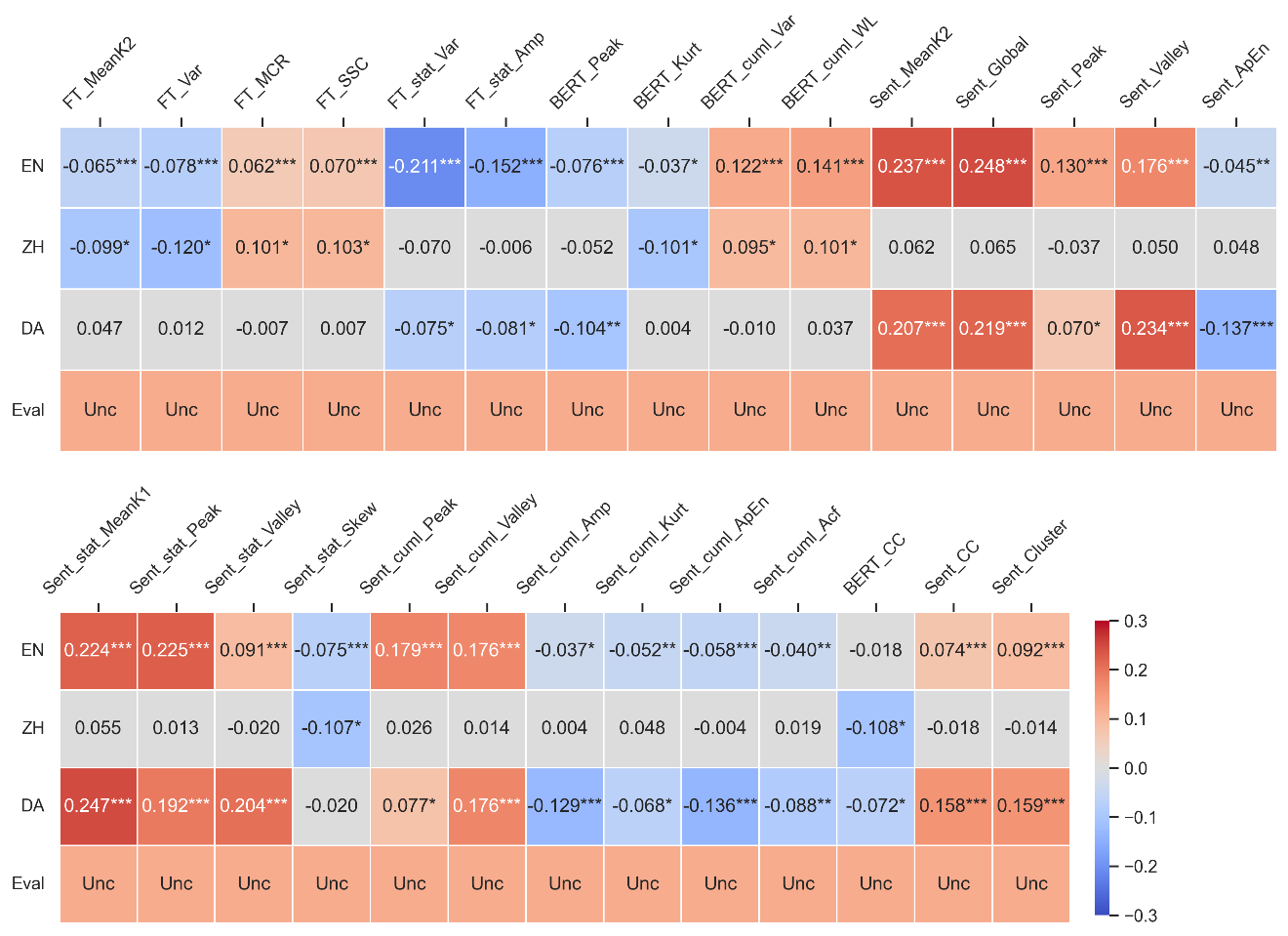


**Fig. S1**. Measures evaluated as uncertain (Unc). The first row indicates the results in the English data (EN), followed by Chinese data (ZH) and Danish (DA) data. Columns refer to semantic measures (on top of each column). Numbers in the cells are Spearman’s correlation coefficients between each measure and coherence in the corresponding dataset. The last row shows the evaluation results, as Pass, Uncertain (Unc), or Fail. *** *q* < 0.001, ** *q* < 0.01, * *q* < 0.05, # *q* < 0. Only correlations with significance level over 0.1 are highlighted, with warm colors for positive ones and cold colors for negative ones.


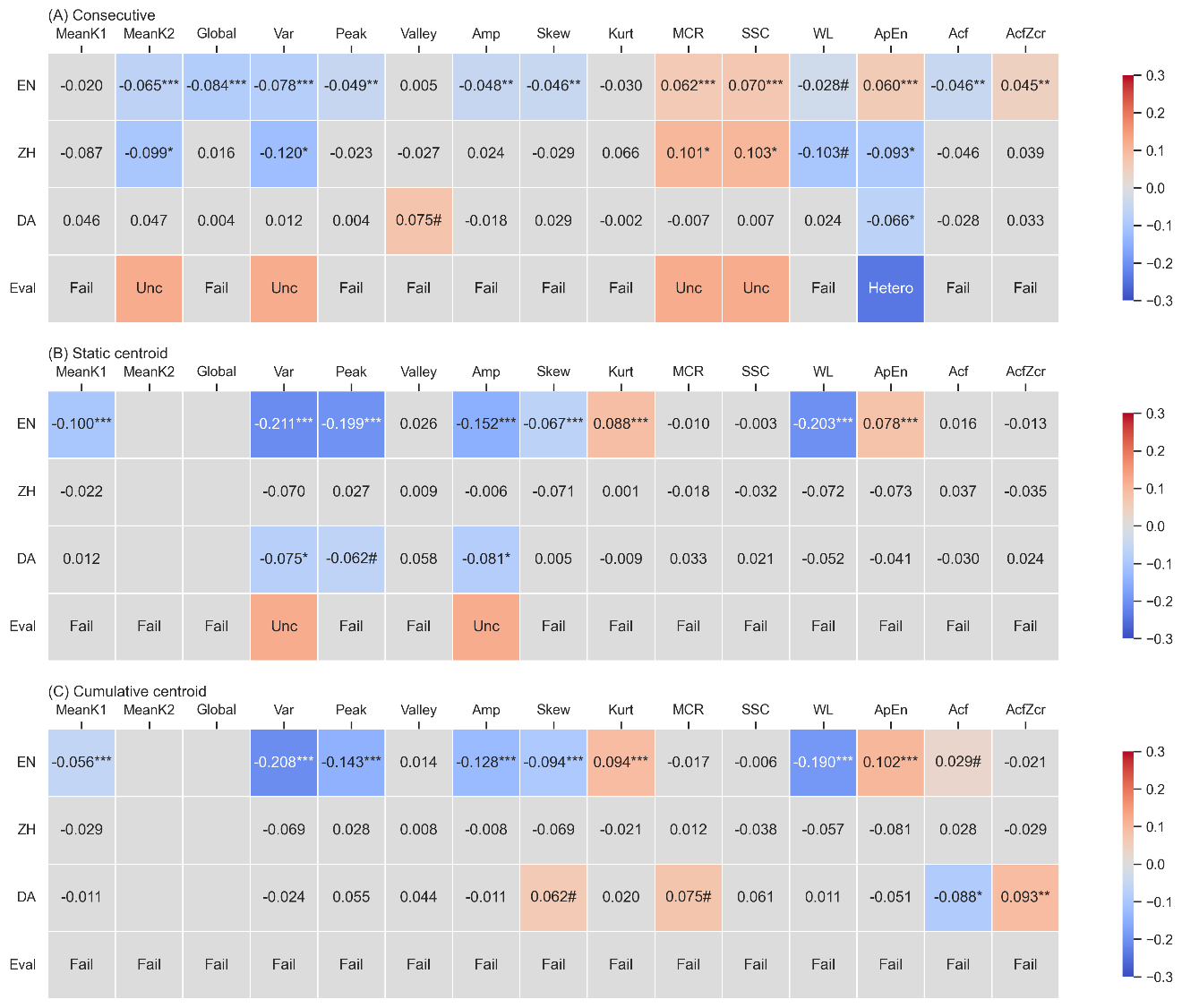


**Fig. S2.** Semantic measures for lexical categories using fastText models. The first row indicates the results in the English data (EN), followed by Chinese data (ZH) and Danish (DA) data. Columns refer to semantic measures (on top of each column). Numbers in the cells are Spearman’s correlation coefficients between each measure and coherence in the corresponding dataset. The last row shows the evaluation results, as Pass, Uncertain (Unc), or Fail. *** *q* < 0.001, ** *q* < 0.01, * *q* < 0.05, # *q* < 0. Only correlations with significance level over 0.1 are highlighted, with warm colors for positive ones and cold colors for negative ones.


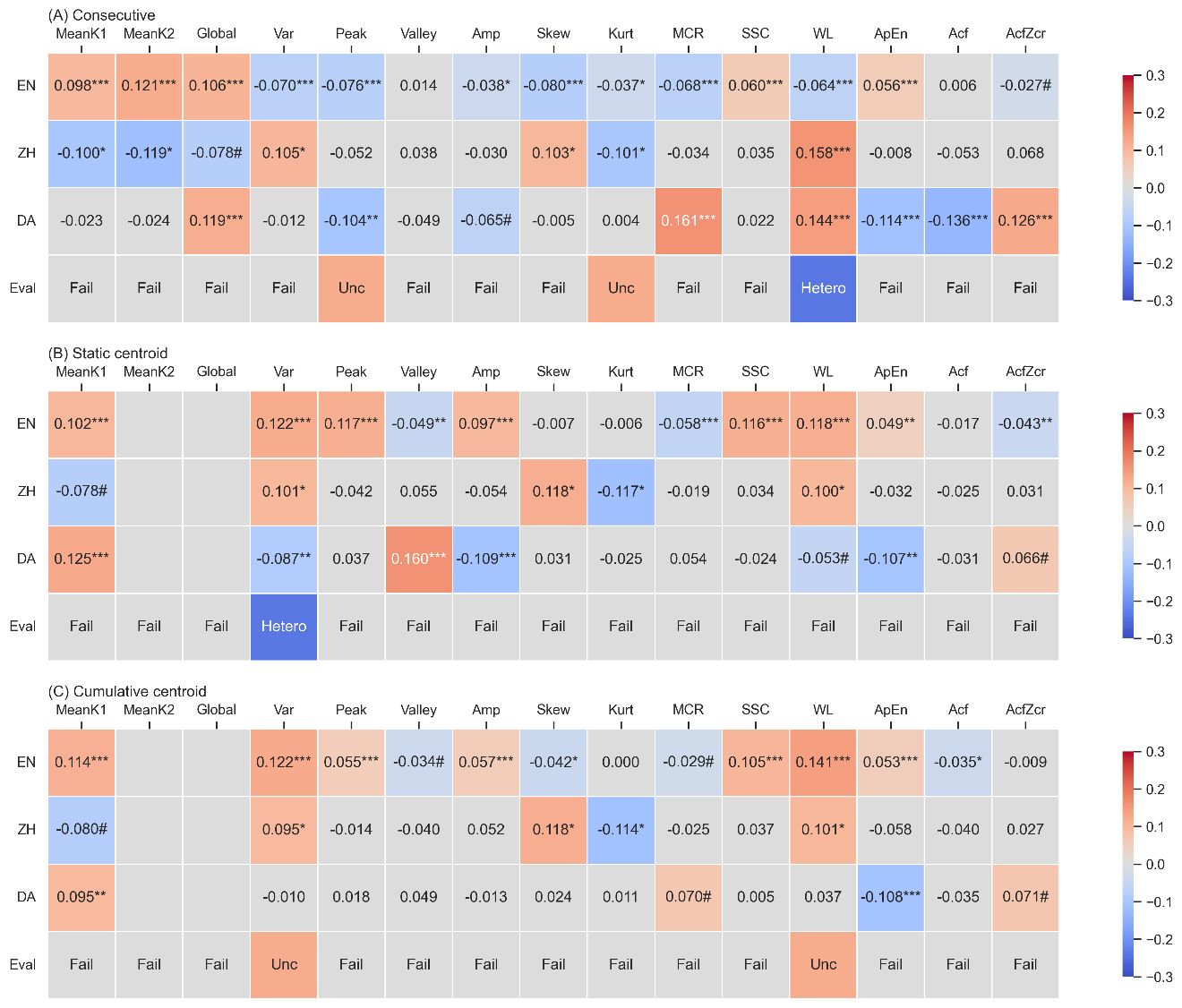


**Fig. S3.** Semantic measures for tokens using BERT models. The first row indicates the results in the English data (EN), followed by Chinese data (ZH) and Danish (DA) data. Columns refer to semantic measures (on top of each column). Numbers in the cells are Spearman’s correlation coefficients between each measure and coherence in the corresponding dataset. The last row shows the evaluation results, as Pass, Uncertain (Unc), or Fail. *** *q* < 0.001, ** *q* < 0.01, * *q* < 0.05, # *q* < 0. Only correlations with significance level over 0.1 are highlighted, with warm colors for positive ones and cold colors for negative ones.


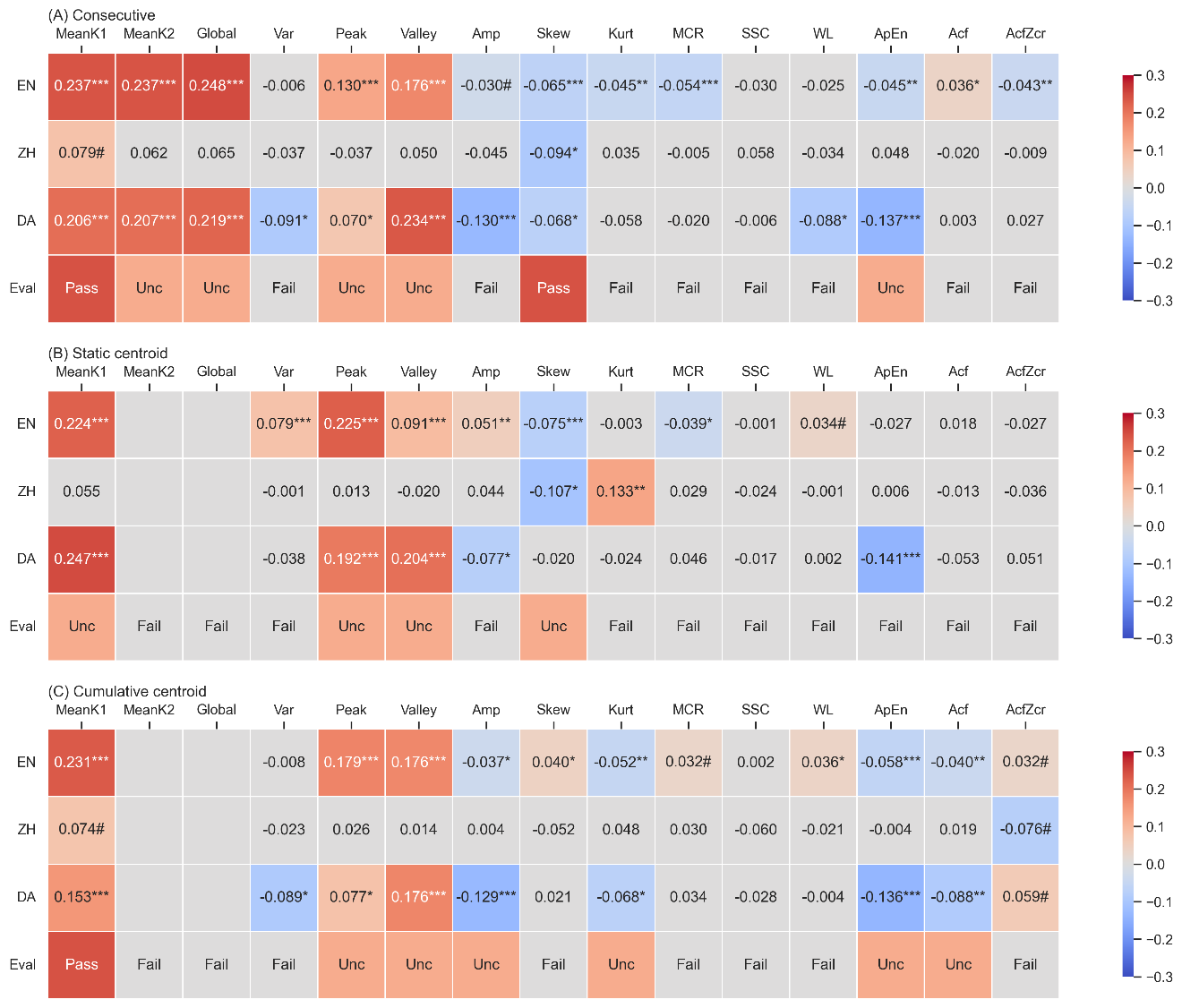


**Fig. S4.** Semantic measures for sentences using sentence transformers models. The first row indicates the results in the English data (EN), followed by Chinese data (ZH) and Danish (DA) data. Columns refer to semantic measures (on top of each column). Numbers in the cells are Spearman’s correlation coefficients between each measure and coherence in the corresponding dataset. The last row shows the evaluation results, as Pass, Uncertain (Unc), or Fail. *** *q* < 0.001, ** *q* < 0.01, * *q* < 0.05, # *q* < 0. Only correlations with significance level over 0.1 are highlighted, with warm colors for positive ones and cold colors for negative ones.


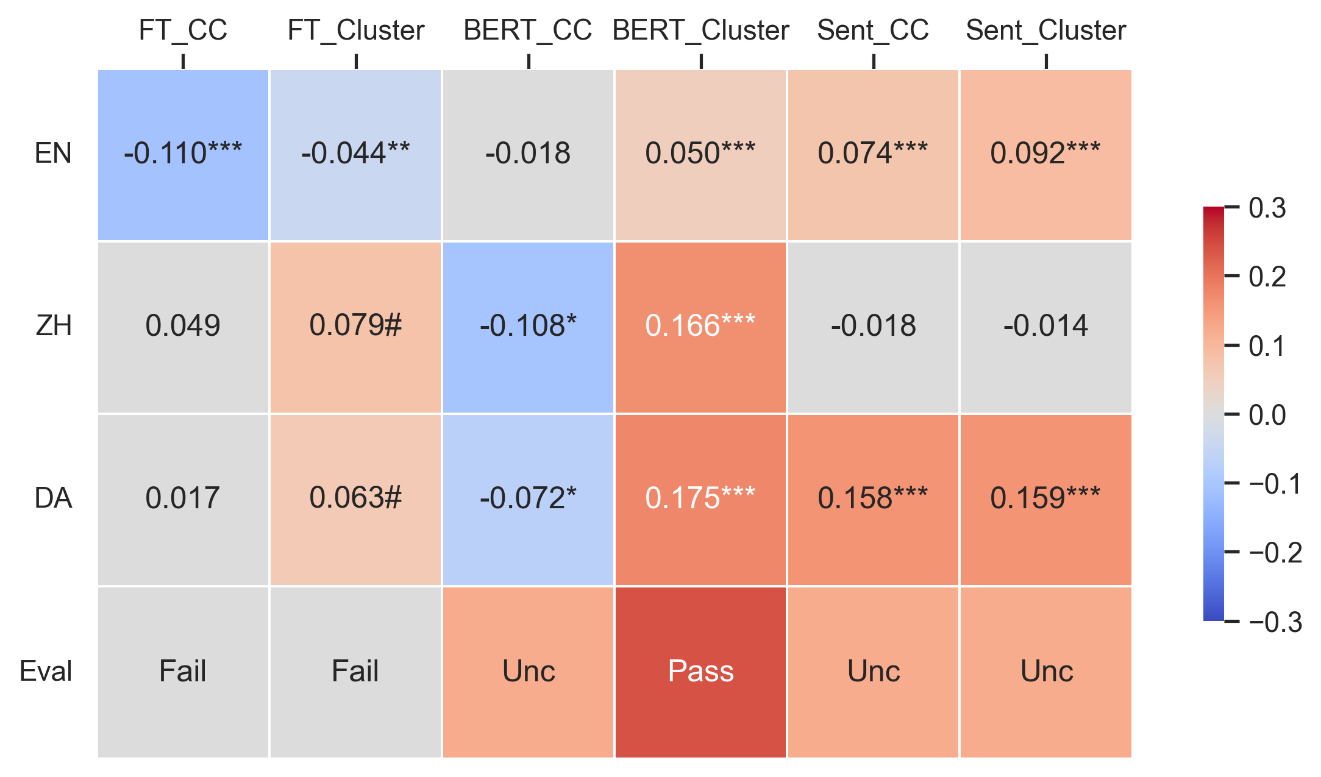


**Fig. S5.** Graph-theoretical measures. The first row indicates the results in the English data (EN), followed by Chinese data (ZH) and Danish (DA) data. Columns refer to semantic measures (on top of each column). Numbers in the cells are Spearman’s correlation coefficients between each measure and coherence in the corresponding dataset. The last row shows the evaluation results, as Pass, Uncertain (Unc), or Fail. *** *q* < 0.001, ** *q* < 0.01, * *q* < 0.05, # *q* < 0. Only correlations with significance level over 0.1 are highlighted, with warm colors for positive ones and cold colors for negative ones.


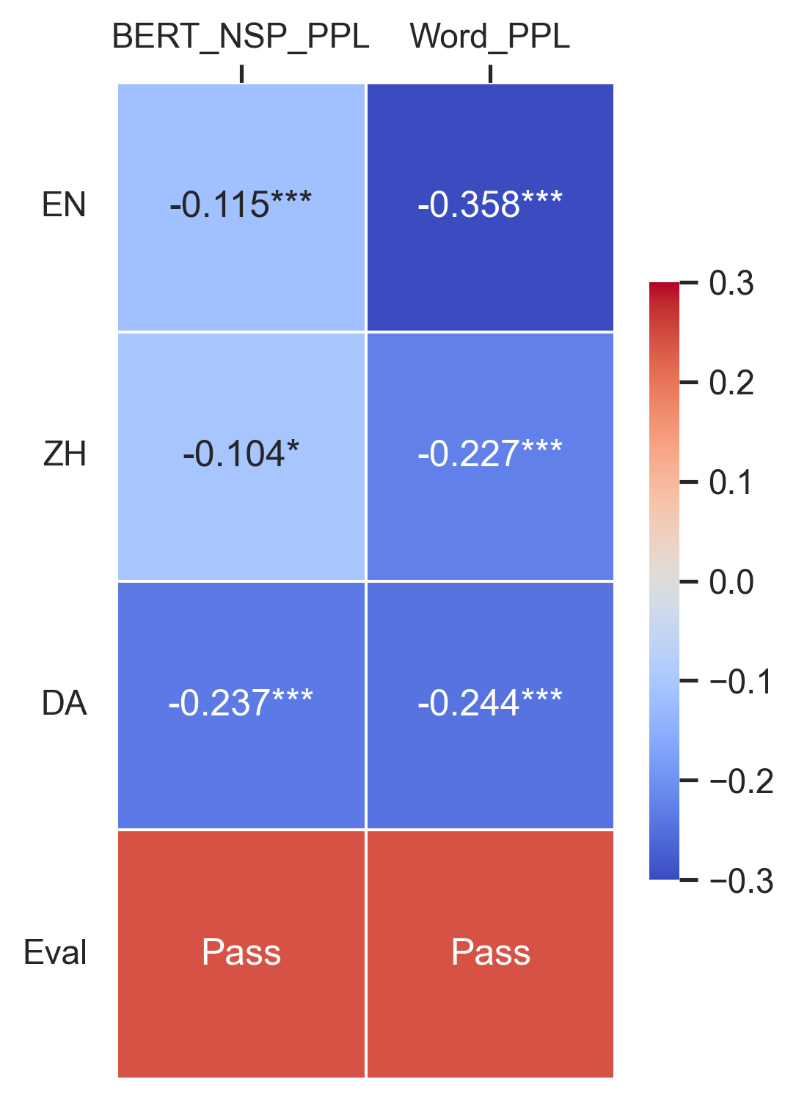


**Fig. S6.** Probability metrics. The first row indicates the results in the English data (EN), followed by Chinese data (ZH) and Danish (DA) data. Columns refer to semantic measures (on top of each column). Numbers in the cells are Spearman’s correlation coefficients between each measure and coherence in the corresponding dataset. The last row shows the evaluation results, as Pass, Uncertain (Unc), or Fail. *** *q* < 0.001, ** *q* < 0.01, * *q* < 0.05, # *q* < 0. Only correlations with significance level over 0.1 are highlighted, with warm colors for positive ones and cold colors for negative ones.

**SI References**

Avants, B. B., Epstein, C. L., Grossman, M., & Gee, J. C. (2008). Symmetric diffeomorphic image registration with cross-correlation: Evaluating automated labeling of elderly and neurodegenerative brain. *Medical Image Analysis*, *12*(1), 26–41. https://doi.org/10.1016/j.media.2007.06.004

Behzadi, Y., Restom, K., Liau, J., & Liu, T. T. (2007). A component based noise correction method (CompCor) for BOLD and perfusion based fMRI. *NeuroImage*, *37*(1), 90–101. https://doi.org/10.1016/j.neuroimage.2007.04.042

Dale, A. M., Fischl, B., & Sereno, M. I. (1999). Cortical Surface-Based Analysis: I. Segmentation and Surface Reconstruction. *NeuroImage*, *9*(2), 179–194. https://doi.org/10.1006/nimg.1998.0395

Fonov, V., Evans, A., McKinstry, R., Almli, C., & Collins, D. (2009). Unbiased nonlinear average age-appropriate brain templates from birth to adulthood. *NeuroImage*, *47, Supplement 1*, S102. https://doi.org/10.1016/S1053-8119(09)70884-5

Greve, D. N., & Fischl, B. (2009). Accurate and robust brain image alignment using boundary-based registration. *NeuroImage*, *48*(1), 63–72. https://doi.org/10.1016/j.neuroimage.2009.06.060

Jenkinson, M., Bannister, P., Brady, M., & Smith, S. (2002). Improved Optimization for the Robust and Accurate Linear Registration and Motion Correction of Brain Images. *NeuroImage*, *17*(2), 825–841. https://doi.org/10.1006/nimg.2002.1132

Klein, A., Ghosh, S. S., Bao, F. S., Giard, J., Häme, Y., Stavsky, E., … Keshavan, A. (2017). Mindboggling morphometry of human brains. *PLOS Computational Biology*, *13*(2), e1005350. https://doi.org/10.1371/journal.pcbi.1005350

Lanczos, C. (1964). Evaluation of Noisy Data. *Journal of the Society for Industrial and Applied Mathematics Series B Numerical Analysis*, *1*(1), 76–85. https://doi.org/10.1137/0701007

Power, J. D., Mitra, A., Laumann, T. O., Snyder, A. Z., Schlaggar, B. L., & Petersen, S. E. (2014). Methods to detect, characterize, and remove motion artifact in resting state fMRI. *NeuroImage*, *84*(Supplement C), 320–341. https://doi.org/10.1016/j.neuroimage.2013.08.048

Satterthwaite, T. D., Elliott, M. A., Gerraty, R. T., Ruparel, K., Loughead, J., Calkins, M. E., … Wolf, D. H. (2013). An improved framework for confound regression and filtering for control of motion artifact in the preprocessing of resting-state functional connectivity data. *NeuroImage*, *64*(1), 240–256. https://doi.org/10.1016/j.neuroimage.2012.08.052

Tustison, N. J., Avants, B. B., Cook, P. A., Zheng, Y., Egan, A., Yushkevich, P. A., & Gee, J. C. (2010). N4ITK: Improved N3 Bias Correction. *IEEE Transactions on Medical Imaging*, *29*(6), 1310–1320. https://doi.org/10.1109/TMI.2010.2046908

Watts, D. J., & Strogatz, S. H. (1998). Collective dynamics of ‘small-world’ networks. *Nature, 393*(6684), Article 6684. https://doi.org/10.1038/30918

Zhang, J., Wang, J., Wu, Q., Kuang, W., Huang, X., He, Y., & Gong, Q. (2011). Disrupted Brain Connectivity Networks in Drug-Naive, First-Episode Major Depressive Disorder. *Biological Psychiatry, 70*(4), 334–342. https://doi.org/10.1016/j.biopsych.2011.05.018

Zhang, Y., Brady, M., & Smith, S. (2001). Segmentation of brain MR images through a hidden Markov random field model and the expectation-maximization algorithm. *IEEE Transactions on Medical Imaging*, *20*(1), 45–57. https://doi.org/10.1109/42.906424
